# Spectroelectrochemical Analysis of the Water Oxidation Mechanism on Doped Nickel Oxides

**DOI:** 10.1021/jacs.1c08152

**Published:** 2022-04-20

**Authors:** Reshma R. Rao, Sacha Corby, Alberto Bucci, Miguel García-Tecedor, Camilo A. Mesa, Jan Rossmeisl, Sixto Giménez, Julio Lloret-Fillol, Ifan E. L. Stephens, James R. Durrant

**Affiliations:** † Department of Chemistry, Centre for Processable Electronics, Imperial College London, London W12 0BZ, U.K.; ‡ Institute of Chemical Research of Catalonia (ICIQ), The Barcelona Institute of Science and Technology, Avinguda Països Catalans 16, 43007 Tarragona, Spain; § Institute of Advanced Materials (INAM), University Jaume I, 12071 Castello de la Plana, Spain; ∥ Department of Chemistry, University of Copenhagen, Universitetsparken 5, Copenhagen DK-2100, Denmark; ⊥ Department of Materials, Royal School of Mines, Imperial College London, South Kensington Campus, London SW7 2AZ, U.K.

## Abstract

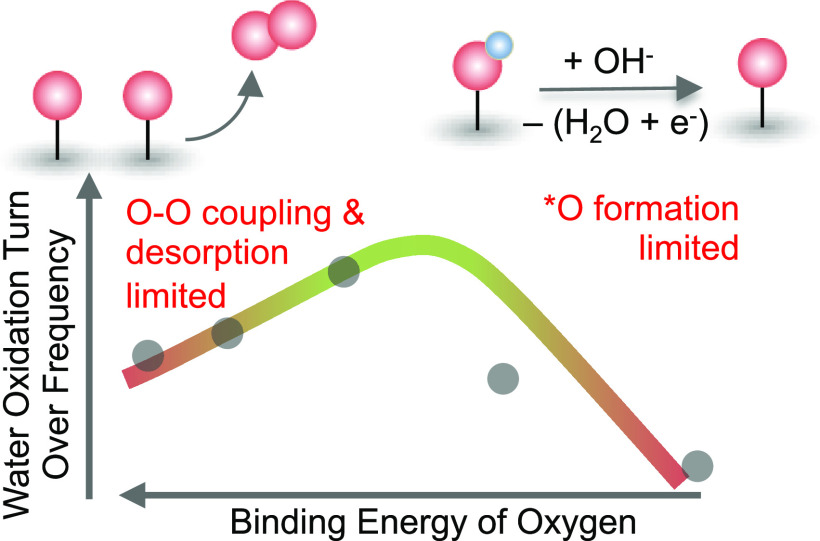

Metal oxides and oxyhydroxides exhibit state-of-the-art activity for the oxygen evolution reaction (OER); however, their reaction mechanism, particularly the relationship between charging of the oxide and OER kinetics, remains elusive. Here, we investigate a series of Mn-, Co-, Fe-, and Zn-doped nickel oxides using *operando* UV–vis spectroscopy coupled with time-resolved stepped potential spectroelectrochemistry. The Ni^2+^/Ni^3+^ redox peak potential is found to shift anodically from Mn- < Co- < Fe- < Zn-doped samples, suggesting a decrease in oxygen binding energetics from Mn- to Zn-doped samples. At OER-relevant potentials, using optical absorption spectroscopy, we quantitatively detect the subsequent oxidation of these redox centers. The OER kinetics was found to have a second-order dependence on the density of these oxidized species, suggesting a chemical rate-determining step involving coupling of two oxo species. The intrinsic turnover frequency per oxidized species exhibits a volcano trend with the binding energy of oxygen on the Ni site, having a maximum activity of ∼0.05 s^–1^ at 300 mV overpotential for the Fe-doped sample. Consequently, we propose that for Ni centers that bind oxygen too strongly (Mn- and Co-doped oxides), OER kinetics is limited by O–O coupling and oxygen desorption, while for Ni centers that bind oxygen too weakly (Zn-doped oxides), OER kinetics is limited by the formation of oxo groups. This study not only experimentally demonstrates the relation between electroadsorption free energy and intrinsic kinetics for OER on this class of materials but also highlights the critical role of oxidized species in facilitating OER kinetics.

## Introduction

Improving the kinetics of electrochemical water oxidation is key to increasing the efficiency of hydrogen production from renewable sources,^1^ production of carbon-neutral fuels such as ethylene,^2^ and rechargeable metal-air batteries.^3^ Metal oxides^4^ and oxyhydroxides^5−8^ exhibit state-of-the-art activity for the oxygen evolution reaction (OER), but fundamental atomic-level insights into the reaction mechanism are often unknown. For kinetically challenging reactions, involving multiple proton and electron transfers, the rate-determining step is commonly identified as the elementary step with the largest barrier.^9,10^ However, probing the rate-determining step for OER on metal oxides/oxyhydroxides is challenging. Density functional theory studies (DFT) typically model the OER reaction as four concerted proton- and electron-transfer steps and compute the thermodynamic barrier for forming *OH, *O, and *OOH intermediates on the active site.^11,12^ Consequently, the potential at which all steps are downhill in free energy is termed as the “limiting potential”.^13^ However, the rate-determining step can be different from the potential limiting step in the following three scenarios (i) when proton- and electron-transfer steps are sequential, resulting in the formation of charged intermediates that are not well modeled by DFT,^14,15^ (ii) if chemical steps, neglected in simple mechanisms proposed by standard DFT calculations, have large barriers,^16,17^ or (iii) when a step, which is more exergonic than the potential limiting step, has a larger barrier than that of the potential limiting step.^18^ Therefore, gaining fundamental understanding of OER mechanisms on oxides relies on understanding the nature of the rate-determining step as well as the role of electrochemical potential in modulating the potential limiting step.

Ni-based electrodes have been used since the late 1800s as catalysts for the OER at the anode, but the mechanism for the reaction is still widely debated.^19^ Currently, transition-metal-doped NiOOH, particularly Fe-doped NiOOH (Ni_*x*_Fe_1–*x*_OOH),^5−8,20−22^ has gained significant attention for their high OER activity. Specifically, for Ni_*x*_Fe_1–*x*_OOH catalysts, the active site has been disputed, with some studies indicating that the active site is Ni, where Fe alters the electronic structure of Ni,^23,24^ while other studies have argued that Fe is the active site.^25−28^ Interestingly, under OER conditions, UV–vis spectroscopy measurements have shown an increase in absorption with applied potential in the OER region, which has been attributed by Görlin et al. to the increase in the density of Ni^4+^ sites,^23^ while Goldsmith et al. have suggested the formation of Fe^4+^ species.^29^ Furthermore, using time-resolved spectroelectrochemistry for three Ni-based oxyhydroxide catalysts with varying Ni/Fe contents, Francàs et al. determined the density of these oxidized species corresponding to the increase in optical absorption and extracted a corresponding intrinsic turnover frequency per oxidized species.^30^ Depending on the active center, DFT calculations suggest that at potentials of ∼1.6 to 1.8 eV, *O species would be more stable on the surface compared to *OH species.^31,32^ This theoretical finding is consistent with the notion that oxidized Ni or Fe states would begin to accumulate at these potentials during OER. However, in addition to the identification of active species, another important consideration is the role of the oxidized states in driving water oxidation, which remains controversial to date.

The influence of oxidized species on OER was first investigated by Conway in the late 1950s, where the potential decay from OER-relevant potentials to open-circuit potentials was accompanied by the evolution of oxygen, suggesting that oxidized states could be reduced to form molecular oxygen.^33^ These results have also been corroborated recently on Ni(*Fe*)OOH, where a decrease in the optical signal has been observed as a function of time during potential decay to open circuit from oxidizing potentials.^30^ Mechanistically, Nocera et al. have proposed that oxo species on adjacent Ni centers can chemically combine to form molecular oxygen,^34^ similar to the mechanism for OER postulated on CoP_i_^35,36^ and CoOOH.^37,38^ This suggests that an increase in the density of oxidized states can increase OER kinetics by facilitating O–O coupling between adjacent oxo groups. Chemical rate-determining steps on metal oxides have also recently gained attention for IrO_*x*_ catalysts where the barrier for chemical water dissociation on the surface was found to be dependent on the coverage of oxidized states because of long-range effects, with the role of electrochemical potential being only to promote the formation of these oxidized states.^16,39,40^ The advent of quantitative spectroscopy coupled with DFT has led to a renewed focus on determining whether chemical or electrochemical steps control electrochemical reactions as well as the applicability of outer-sphere electron-transfer theories to model inner sphere reactions.^39,40^

Here, we study the mechanism of water oxidation on a series of high-performance Mn-, Co-, Fe-, and Zn-doped NiO catalysts. These oxide-based catalysts have recently demonstrated promising activity and stability and can be synthesized using low-cost and scalable processes such as solution combustion.^41^ Using operando UV–vis spectroscopy and stepped potential spectroelectrochemistry, we detected an increase in the density of oxidized species at oxygen evolution potentials, attributable to oxidized Ni^4+^ species. The dopants are shown to influence the potential at which oxidized species are formed, in the following order: Mn- < Co- < Fe- < undoped < Zn-doped NiO_*x*_, thus demonstrating their influence on the oxygen binding energetics. The intrinsic turnover frequency (TOF) per oxidized Ni^4+^ site is found to be the largest for the Fe-doped sample followed by the Co- and Mn-doped samples and then by the undoped and Zn-doped samples at 1.65 V_RHE_. By correlating the TOF with the density of oxidized species, we find that the OER has a second-order dependence on the density of these Ni^4+^species. Consequently, we propose a reaction mechanism based on a potential determining step of the formation of Ni^4+^(Ni-OO) species from Ni^3+^(Ni-OOH) followed by a chemical rate-determining step involving the coupling of two oxo species to form molecular oxygen. Based on this mechanism, we elucidate the role of metal dopants in modulating OER activity and provide insights into the rational design of more active catalysts. This study thus reveals a direct experimental methodology to probe chemical rate-determining steps on a series of doped NiO and understand its influence on OER kinetics.

## Results and Discussion

### Precatalytic Oxidation of Ni

**Figure 1:**
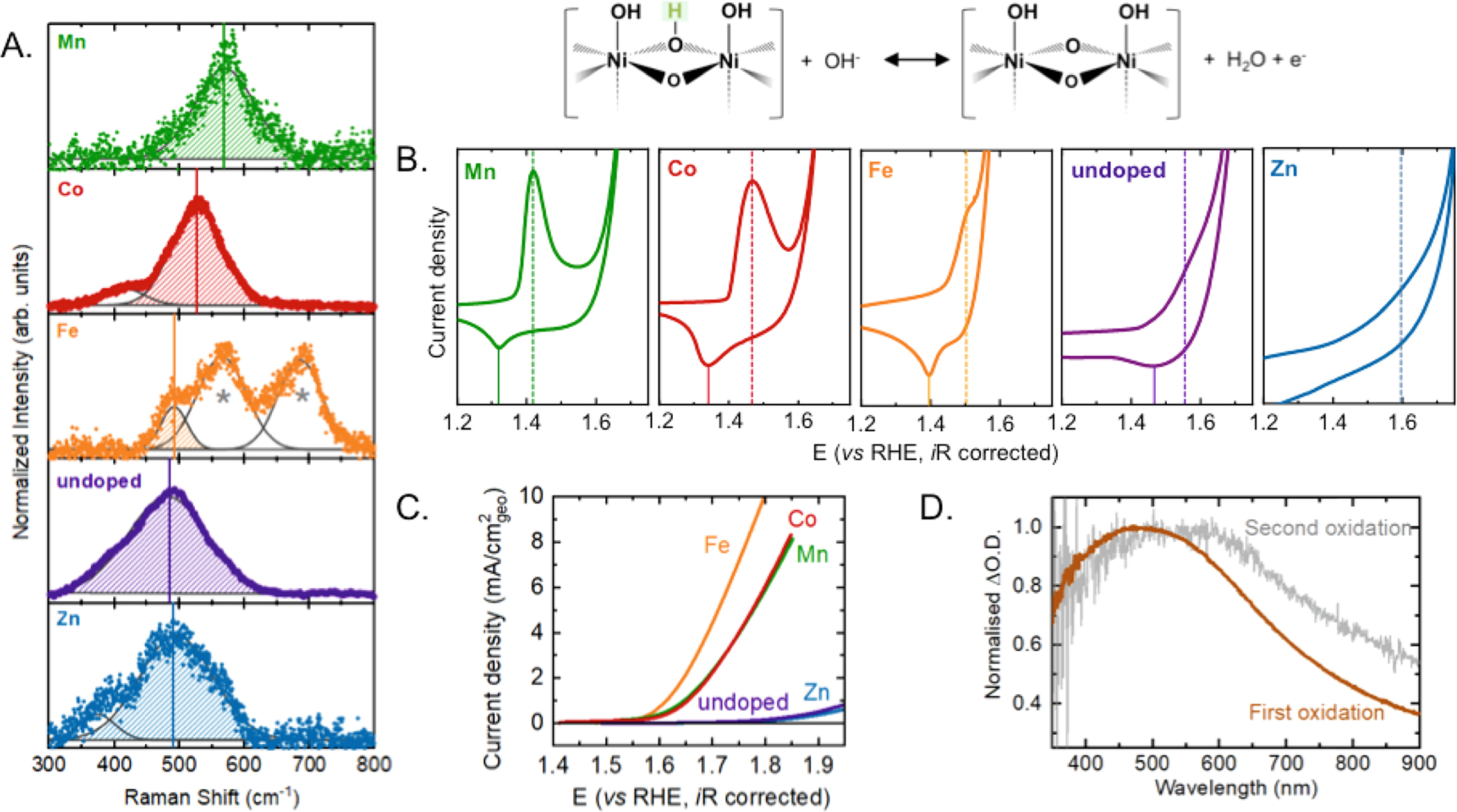
(A) Raman spectra measured at open-circuit potential for the 10% Mn-doped (green), Co-doped (red), Fe-doped (orange), undoped (purple), and Zn-doped (blue) NiO. The shaded area indicates the stretching frequency of the Ni–O. Peaks corresponding to FeOOH in the Fe-doped sample are indicated by *. (B) Cyclic voltammograms of the Ni^2+^/Ni^3+^ redox peak position for the chemistries investigated with a schematic description of the redox process shown above. Mn-, Co- and Fe- shift the redox peak cathodically and Zn-shifts it anodically relative to the undoped sample. All measurements were made in Fe-free 0.1 M KOH on samples deposited on FTO (loading = 2.5 mg/cm^2^) at 10 mV/s. (C) Linear sweep voltammograms of M_0.1_Ni_0.9_O (M = Fe (orange), Co (red), Mn (green), Zn (blue), and undoped NiO (purple)). (D) Representative normalized differential absorption for the species corresponding to the first redox transition (brown, obtained from the difference between the spectra at the open-circuit potential before and after activation) and the second redox transition, generating the oxidized species present at OER potentials (gray, obtained from the difference between spectra taken at 10 mV intervals in the potential range where a current density of ∼1 mA/cm^2^ was recorded) for the Mn-doped NiO.

Metal-doped nickel oxides (M_0.1_Ni_0.9_O, M = Mn, Co, Fe, and Zn) were prepared by solution combustion synthesis, as described previously.^41^ The doping concentration was confirmed using inductively coupled plasma mass spectrometry, and all pristine oxides were present in the rock salt structure, as confirmed by X-ray diffraction (XRD) (Figure S1). The as-synthesized oxides had a foamy morphology (Figure S2) and particle sizes ranging from ∼15 to 70 nm (Figure S3, Table S1). Raman spectra collected under open-circuit conditions were used to provide further information about the as-synthesized materials (Figures 1A and S4). First-order Raman scattering is forbidden in the perfect rock-salt cubic structure of NiO and is absent in single crystals, but more disordered and defect-rich nanoparticles exhibit Raman bands below 600 cm^–1^, which can be assigned to one-phonon (1P) Ni–O modes (transverse optical and longitudinal optical).^42^ The presence of these modes between ∼470 and 600 cm^–1^ in the nanoparticles synthesized using solution combustion (Figure 1A) indicates a significant degree of structural defects in these samples. These defects could result in sites with a range of different local structural and chemical environments. In addition to the Ni–O stretching mode, the Fe-doped sample also exhibits two additional bands at ∼570 cm^–1^ and ∼660 nm^–1^ which are characteristic of γ-FeOOH^43,44^ and has been observed previously on Fe-doped NiOOH.^45^ In any case, the γ-FeOOH should be a minor component of the bulk because the characterization of the samples before and after exposure to electrocatalytic conditions showed the rock salt as the only detectable phase.^41^ The Ni–O stretching frequency in the Mn- (∼570 cm^–1^), Co- (∼527 cm^–1^) and Fe- (∼490 cm^–1^) and Zn-doped samples (∼490 cm^–1^) are blue-shifted compared to that in the undoped sample, ∼480 cm^–1^. The blueshift of the Ni–O stretching frequency has been previously attributed to structural defects and consequently an increase in the density of oxidized Ni^3+^ species in NiO.^46−48^ Therefore, we can hypothesize that the dopants increase the density of oxidized Ni sites, and this effect decreases from the Mn-doped NiO to the Co-, Fe-, undoped, and Zn-doped oxide. This is in agreement with our previous observations, where X-ray photoelectron spectroscopy (XPS) detected an increase in Ni^3+^ at the surface in doped NiO electrodes compared with the undoped ones and transmission electron microscopy (TEM), scanning tunneling electron microscopy (STEM), PXRD, and extended X-ray absorption fine structure (EXAFS) indicated that the Fe, Mn, and Co present more structural defects.^41^

The role of the dopants in altering the electronic structure of the Ni center is also evident from the electrochemical redox potential of Ni centers in these doped oxides. *Operando* Raman spectroscopy reveals that all oxides are present in the oxyhydroxide phase once they have been exposed to water oxidation conditions (potentials greater than ∼1.7 V_RHE_), identified by the peaks at ∼550 cm^–1^ (polarized A_1g_ stretching mode) and ∼470 cm^–1^ (depolarized *E*_g_ bending mode)^49,50^ of Ni–O in NiOOH, Figure S5. Previous studies have noted shift of the Raman peaks in the presence of different cations in solution (Li^+^, Na^+^, K^+^, Cs^+^),^51^ but no peak shift in the presence of D_2_O relative to H_2_O,^52^ suggesting the presence of oxidized Ni sites where the adsorbed *O can interact with cations in the electrolyte. Cyclic voltammograms obtained in Fe-free 0.1 M KOH (pH ∼13) at a scan rate of 10 mV/s (Figure 1B) show the presence of a redox peak prior to the evolution of oxygen. This redox process has been attributed to hydroxide-mediated deprotonation involving the oxidation of a Ni site from +2 to +3, Ni(OH)_2_ + OH^–^ → NiOOH + H_2_O + e^–^.^50^ The redox peak position is shown to depend on the dopant, centered at ∼1.35 V_RHE_ for Mn-doped, ∼1.4 V_RHE_ for Co-doped, ∼1.45 V_RHE_ for Fe-doped, and ∼1.5 V_RHE_ for the undoped NiO (Figures S6 and S7). Although the redox peak center for the Zn-doped NiO is not distinctly evident, subtracting the first scan from the 50^th^ scan shows the evolution of a redox feature at ∼1.62 V_RHE_. Our previous work suggests that while trace Zn leaching is observed after long-term testing (4.6% of the initial Zn content after holding the electrodes at 10 mA/cm^2^_geometric_ for 24 h followed by 2500 cyclic voltammograms), resulting in the deviation of the surface Zn composition from the as-synthesized materials, the activity is not altered.^41^ Here, we note that the redox peak on the synthesized undoped NiO is at higher potentials compared to Ni(OH)_2_ films^8,53^ but are comparable to reports on magnetron-sputtered NiO_*x*_ thin films^54^ and NiO nanoparticles annealed at high temperatures.^46^ Doping NiO with Fe also induces a cathodic shift of the redox peak, which is different to the trend observed on Fe-doped Ni(OH)_2_ films,^8,21^ further suggesting that the oxide-derived catalysts exhibit different redox properties to those synthesized as hydroxides. Because this redox transition corresponds to the first deprotonation of a hydroxyl group bound to the metal center, Ni-(OH)**OH** + OH^–^ → Ni-(OH)**O** + H_2_O + e^–^ (see Figure 1 illustration), the free energy change corresponding to the reaction is given by Δ*G*_1_*=* Δ*G*_O1_ – Δ*G*_OH1_ – e*U*_RHE_, where Δ*G*_O1_ and Δ*G*_OH1_ are the free energy of adsorption of *O and *OH at the bridging site, and U_RHE_ is the potential.^55^ Consequently, this experimentally measured redox peak potential can be directly related to the [Δ*G*_O_ – Δ*G*_OH_], which has traditionally been used as a computational activity descriptor.^11,12^ While the exact value of the redox peak position can also be influenced by factors such as the pH^53^ and ions in solution,^51^ here, we only change the oxide composition, and we can thus make meaningful comparison of the trends in the redox peak position observed. Therefore, here, we find that the oxidizing potential of Ni is altered by the dopant in the order Mn- < Co- < Fe- < undoped < Zn-doped. The trend in the redox peak potential correlates with the Raman measurements under open-circuit conditions, which show that the degree of defects and density of Ni^3+^ sites decrease from the Mn-doped to the Zn-doped oxide.

### Quantifying Density of Oxidized Species at OER-Relevant Potentials

**Figure 2:**
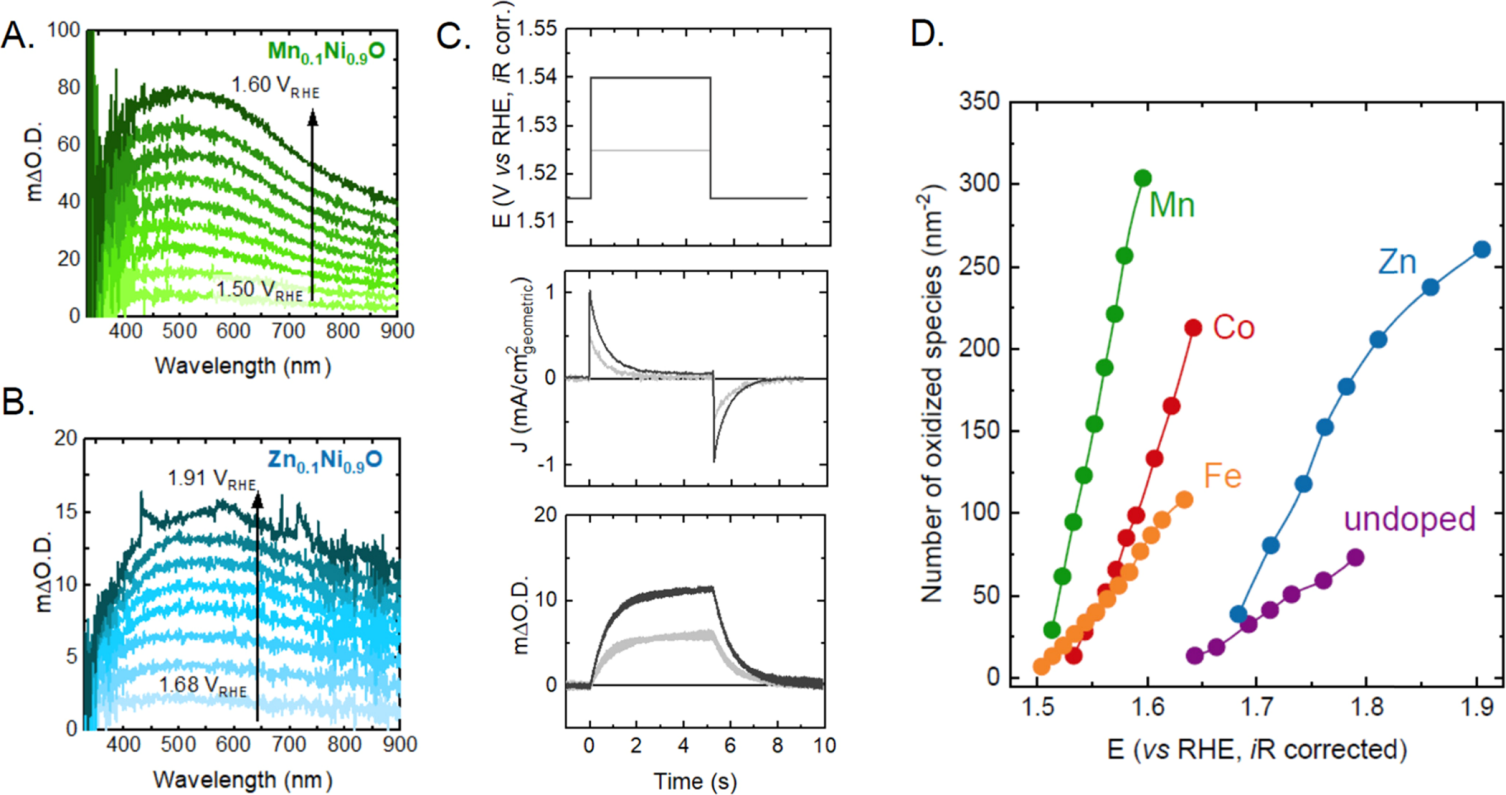
*Operando* UV–vis spectra as a function of potential for (A) Mn_0.1_Ni_0.9_O and (B) Zn_0.1_Ni_0.9_O in Fe-free 0.1 M KOH. The increase in intensity is much larger for the Mn-doped sample and the onset potential for accumulation of oxidized species is also lower (Figure S10 shows data for other samples). (C) Representative stepped potential spectroelectroelectrochemistry measurements for Mn_0.1_Ni_0.9_O showing the applied potential steps (top panel), measured current density (middle panel), and optical signal (bottom panel) as a function of time. (D) Number of oxidized species accumulated per nm^2^ of the geometric surface area for all M_0.1_Ni_0.9_O samples as a function of potential, obtained using steady-state UV–vis spectroscopy measurements. Changes in the optical signal were converted to the density of oxidized species using stepped potential spectroelectrochemistry.

Increasing the potential beyond ∼1.5 V_RHE_ leads to a sharp rise in current density from the evolution of molecular oxygen. Linear sweep voltammograms show that the activity differs significantly for different samples following the order Fe- > Co- ∼ Mn- > undoped ∼ Zn-doped NiO, Figure 1C. In order to probe the mechanism for OER and explain the activity trends, *operando* UV–vis spectroscopy measurements were performed. Using UV–vis spectroscopy, two distinct optical features are apparent with increasing potential. Representative normalized differential absorption (ΔO.D.) spectra for the Mn-doped sample are shown in Figure 1D (Figures S8 and S9 show data for other samples). The first differential spectra observed from the redox peak in the CV and prior to the onset of OER, obtained from the difference between the spectra at the open-circuit potential before and after activation (brown trace, Figure 1D), have a peak centered around ∼500 nm, which we assign to the Ni^2+^/Ni^3+^ redox transition. A similar increase in optical absorption observed at ∼450 nm for Ni-based OER catalysts^23,29,30,54,56^ has been assigned to nickel d–d interband transitions.^57^ Interestingly, upon increasing the potential to the water oxidation regime, a different spectral shape was observed (obtained from the difference between spectra taken at 10 mV intervals in the potential range where a current density of ∼1 mA/cm^2^ was recorded) with a broader peak between ∼400 and 600 nm (gray trace, Figure 1D), suggesting further oxidation of the Ni center. The shape of this spectral feature, particularly the wavelength for maximum absorption, is independent of the dopant (Figure 2A, B). The wavelength for maximum absorption is similar to that reported in previous work on NiO_*x*_ films^54^ and pure NiOOH,^30^ but higher than that obtained for pure FeOOH^30^ and Fe-doped NiOOH, with dopant concentrations of 25%^29^ and 75%, respectively.^30^ This suggests that the increase in absorption is due to the oxidation of Ni centers. Therefore, this second redox process can be attributed to the formation of oxo species from remaining surface hydroxyl groups in the oxyhydroxide structure, NiOOH + OH^–^ → NiOO + H_2_O + e^–^, where these oxo species can have a negative charge.^51,53^ Near-edge X-ray absorption spectroscopy (XAS) measurements also show the presence of highly oxidized Ni centers (>3+) under OER conditions,^23,58−60^ supporting this assignment. Notably, while, it is not possible to detect this redox transition using cyclic voltammetry due to the OER electrocatalytic wave, the presence of this redox process can be observed using operando UV–vis spectroscopy.

By distinguishing the spectral fingerprints of the two oxidized species using optical spectroscopy, we can accurately track the evolution of the second oxidized species in the water oxidation regime. To quantify the density of these species, stepped potential spectroelectrochemistry was performed at 500 nm. A potential step was applied in a potential region where only the second Ni oxidation process is expected (Figure 2C, top panel), and the current density (Figure 2C, middle panel) and optical change (Figure 2C, bottom panel) resulting from this potential step were tracked as a function of time. Upon increasing the potential, an increase in absorption was detected, which returned to its original value when the applied potential was stepped down. The total charge stored in the oxide corresponding to this optical change was quantified by integrating the reduction current while decreasing the potential, as has been shown in our previous work.^30^ Therefore, the charge extracted can be used to provide insight into the density of oxidized species formed, assuming that the formation of each oxidized species involves one electron transfer. By correlating the absorption signal with the charge extracted (Figures S11–S18), the extinction coefficient of these species can be computed^30^ to determine their density as a function of potential, as shown in Figure 2D. The density of oxidized Ni centers is less than 1% of the total Ni centers in the film, which is consistent with previous work that suggests that OER on these materials is confined to the surface layer.^20,54^ Therefore, based on these measurements, the optical intensity at a given potential is converted to a species density (Figure 2D). Interestingly, the onset potential for the formation of these species follows the same trend as the Ni^2+^/Ni^3+^ redox peak position, being the lowest for the Mn-doped oxide, followed by the Co-, Fe-, undoped, and Zn-doped oxide. This finding validates previous theoretical results that show a linear correlation between the Gibbs free energy of the first and second oxidation of the same Ni site, that is, between Δ*G*_1_*=* Δ*G*_O1_ – Δ*G*_OH1_ (Ni(OH)_2_ + OH^–^ → NiOOH + H_2_O + e^–^) and Δ*G*_2_*=* Δ*G*_O2_ – Δ*G*_OH2_ (NiOOH + OH^–^ → NiOO + H_2_O + e^–^).^61^ While these linear free energy scaling relationships have been well documented in the theoretical community,^11,62,63^ this study experimentally demonstrates its applicability to a range of oxides by directly probing these oxidized species in the water oxidation regime.

**Figure 3:**
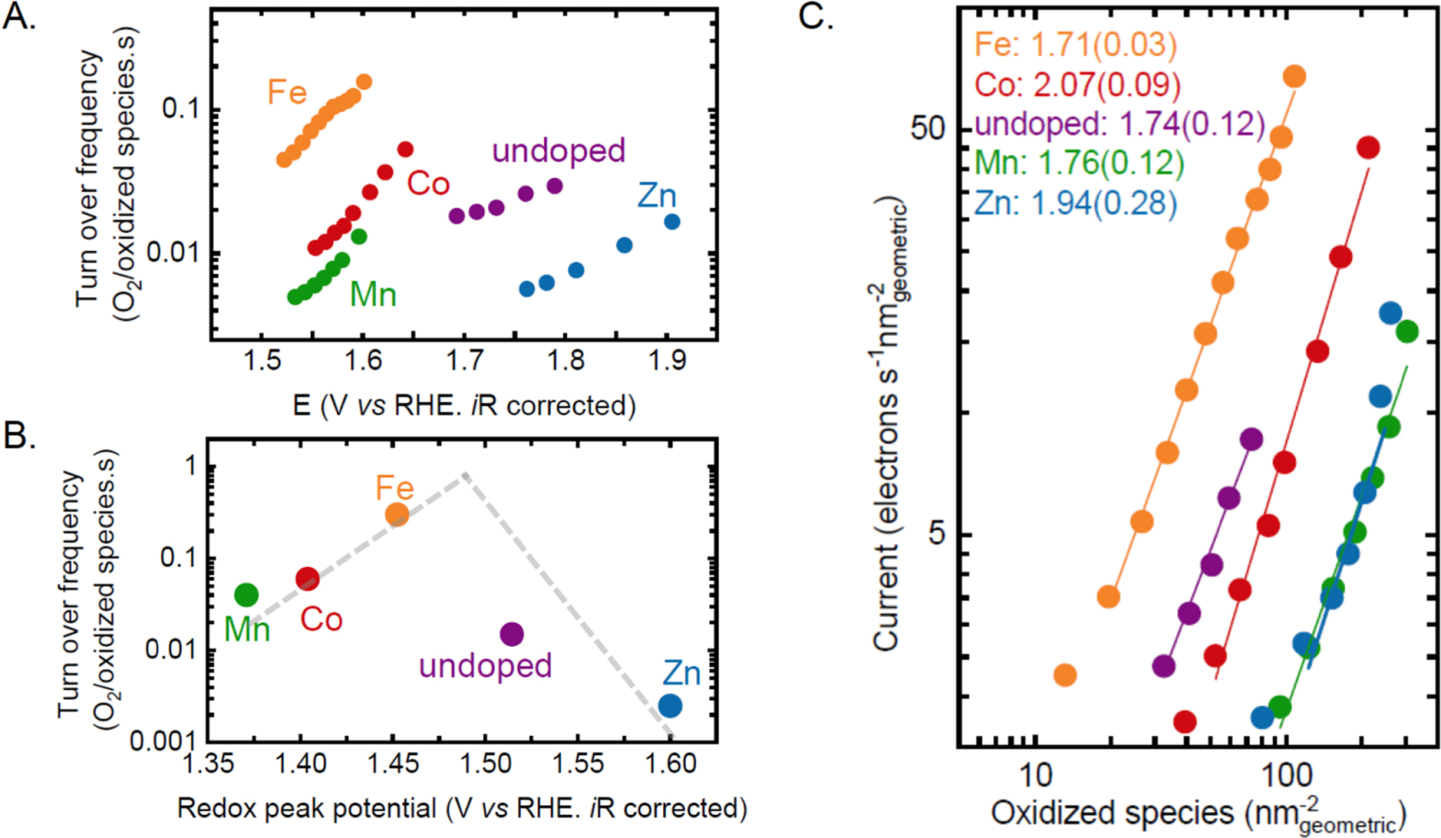
(A) Intrinsic OER activity defined as the TOF per oxidized species per second. The Faradaic efficiency for water oxidation has been assumed to be 100%. (B) Intrinsic turnover frequency measured at 1.65 V_RHE_ plotted as a function of the average redox peak potential of the pre-OER redox peak, except in the case of the Zn-doped sample, where the anodic redox peak center has been used. (C) Log–log plot of the current density as a function of the density of oxidized species. A linear trend with a slope of 2 is observed for all the dopants across the measurement range. The numerical value of the slope and the uncertainty in its value are shown in parenthesis adjacent to the dopant metal.

Based on the quantification of the density of oxidized Ni species as a function of potential, the intrinsic TOF (i.e., number of O_2_ molecules evolved per oxidized state per second) can be determined, Figure 3A. In contrast to most previous experimental studies that report TOFs normalized to a constant density of active sites in the sample (defined either as the total number of metal sites in the bulk, metal sites on the surface, or electrochemically active metal sites), in this work, we use the experimentally measured potential-dependent density of oxidized species to calculate the intrinsic TOF. Notably, the time scale for charge extraction through the film is significantly faster than the water oxidation rate (Figures S11 and 12), and is therefore not a limiting factor for the water oxidation kinetics. The intrinsic TOF per oxidized species, at a given potential, depends on the dopant, being the largest for the Fe-doped material, followed by Co-, Mn-, undoped, and Zn-doped NiO. The TOF at an overpotential of 300 mV for the most active Fe-doped sample is ∼0.05 s^–1^. This value of TOF obtained per oxidized species is comparable to exfoliated NiFe layered double hydroxides (∼0.05 s^–1^),^64^ electrodeposited NiFeOOH deposited on glassy carbon substrates (∼0.05 s^–1^),^65^ and solution-cast NiFeO_*x*_ thin films^66^ (∼0.21 s^–1^) at pH 14, where the activity was normalized to all metal ions in the film. It is slightly higher than that reported in our previous work on electrodeposited Ni(*Fe*)OOH films (∼0.01 s^–1^)^30^ at pH 13 where, similar to this work, the density of oxidized species was used for normalization. Notably, the TOF is lower than the state-of-the-art 5.4 nm NiFe nanoparticles, for which a lower estimate of 1.2 s^–1^ was obtained, assuming all sites in the bulk were active and an upper estimate of 6.2 s^–1^ was obtained assuming only surface sites were active.^20^

### Experimental Volcano Plot Analysis for Water Oxidation

The intrinsic activity for this series of samples can be correlated to the binding energetics of oxygenated intermediates on the Ni site, experimentally measured by the redox peak center for the Ni^2+^/Ni^3+^ redox transition. The redox peak potential, an experimental proxy of the theoretically computed oxygen binding energetics (Δ*G*_O_ – Δ*G*_OH_), has been used to explain the activity for IrO_2_(110) epitaxial thin films^67^ and oriented RuO_2_ thin films^68^ as a function of pH for oxygen evolution, as well as molecular nitrogen coordinated metal centers for oxygen reduction.^69^ Similarly, the binding energy of *OH was monitored by changes in the redox peak position on Pt(111) alloyed with subsurface Cu in acid^70^ and base,^71^ and these changes in the redox peak position were correlated to the oxygen reduction activity. Extending these studies beyond single crystals and molecular catalysts is challenging because of the accurate measurement of the intrinsic TOF. Here, we find a “volcano” relationship between the intrinsic TOF at 1.65 V_RHE_ and the redox peak potential, with the Fe-doped sample having the highest activity (Figure 3B). Relative to undoped NiO, doping with 10% Fe results in a cathodic shift of the redox potential by ∼60 mV and an increase in activity by an order of magnitude. A further decrease in the redox peak potential upon doping with 10% Co or Mn results in a decrease in activity relative to the Fe-doped sample. Thus, using this series of doped nickel oxides, we show how the binding energy of oxygenated species can be probed using redox peak potentials from cyclic voltammetry and how this value can be used as an experimental descriptor for the OER activity. Notably, the best-performing catalyst in our study has a Ni^2+^/Ni^3+^ redox peak center of ∼1.45 V_RHE_. As discussed above, the Fe-doped NiO studied here does not have the highest intrinsic TOF reported to date. Some NiFe films and nanoparticles have been reported to have minimum TOF (normalized to all bulk metal sites) of 0.2 and 1 s^–1^, respectively, at a potential of 1.53 V_RHE_.^20,66^ This observation suggests that we have not realized the peak activity in this study. Materials that exhibit intermediate binding energy between the undoped and Fe-doped sample are likely to have a larger TOF.

Mechanistically, experimental validation of Sabatier analysis had been largely confined to single crystal surfaces or molecular catalysts, where the active center density and the local structure were well-defined, and the intrinsic activity per site could be determined. In fact, for oxyhydroxides, Boettcher et al. found that ∼30% doping of NiO_*x*_H_*y*_ with Mn, La, Ti, and Ce resulted in significant changes in the redox peak potential, being the lowest in the case of Mn and the highest for Ce. However, no trend was found between the Ni^2+^/Ni^3+^ redox peak potential and OER activity, possibly because the authors use the Ni^2+^/Ni^3+^ redox peak area to determine the density Ni centers, as opposed to the methodology used in this work, which can directly determine the density of oxidized states at water oxidation potentials.^21^ We also find that the trend between the current density normalized to the geometric area and the redox peak center does not show a pronounced volcano trend (Figure S19) because samples with a lower TOF such as Mn-doped can have relatively large current densities owing to the large density of oxidized species. Recent studies have shown that while bulk metal centers can participate in the Ni^2+^/Ni^3+^ redox, water oxidation is limited to the surface layers of the catalyst,^20,54^ further justifying the need to directly measure the density of oxidized species under water oxidation conditions. By accounting for the different densities of oxidized species and their potential dependence, we have experimentally demonstrated the Sabatier volcano trend for water oxidation on complex oxides. This highlights the importance of directly probing the density of active species to determine intrinsic activity metrics in order to understand OER kinetics.

**Figure 4:**
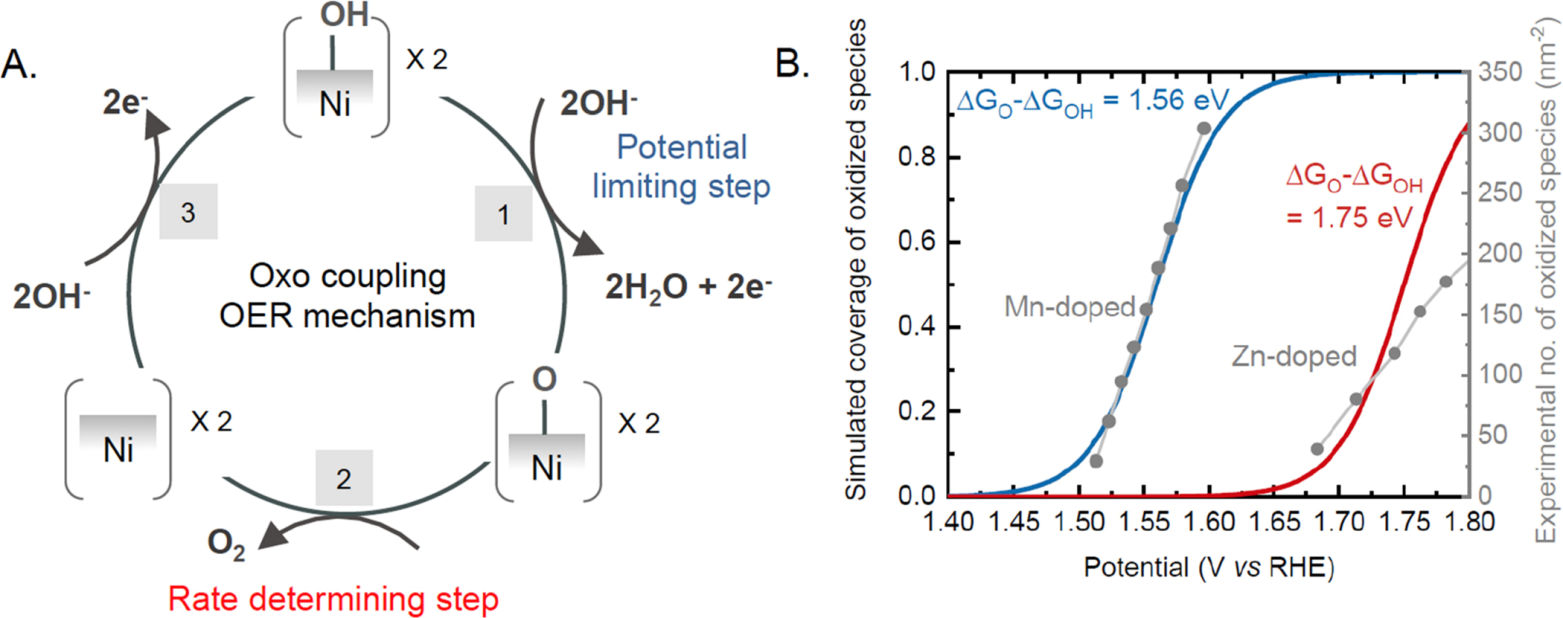
(A) Proposed mechanism for OER involving two adjacent oxidized Ni sites. The potential determining step involves the formation of oxidized species via the deprotonation of *OH groups on the Ni site, and the rate-determining step involves the combination of two adjacent *O groups to form molecular oxygen. (B) Coverage of the oxidized species as a function of potential for a strong-binding Ni site (Δ*G*_O_ – Δ*G*_OH_ = 1.56 eV, blue) and a weak-binding Ni site (Δ*G*_O_ – Δ*G*_OH_ = 1.75 eV, red) obtained using a Langmuir electroadsorption model. Experimental values for the density of oxidized species on the Mn- and Zn-doped samples have been coplotted in gray. For the strong-binding site, the reaction rate would be limited by the rate-determining step involving O_2_ formation, whereas for the weak-binding site, the reaction rate would be limited by the formation of oxidized species.

In order to gain deeper mechanistic insight, we perform a rate-law analysis to determine the relation between the current density and the density of oxidized species. The kinetics for water oxidation on localized, molecular active sites on a number of (photo)electrocatalysts^30,72−74^ has been shown to depend primarily on the density of oxidized species, and the role of the electrochemical potential primarily is to drive the formation of these oxidized species. In this “population” model of water oxidation kinetics, the steady-state current is given by J = k*[*Oxidized species*]^α^, where *k* is the water oxidation rate constant independent of potential and the density of oxidized species, and α is the order of the reaction with respect to the density of oxidized species. The water oxidation activity is not strongly dependent on pH, increasing by only a factor of 2 for a 10-fold increase in the concentration of OH^–^ ions, and thus, the concentration of OH^–^ ions has not been explicitly included in our rate-law analysis (Figures S20 and S21). Consequently, a log–log plot of the current versus the density of oxidized species yields a straight line with the slope corresponding to the numerical value of α. Indeed, a straight line was observed for all the materials considered herein over a one order magnitude of current density, with a constant slope of ∼2 (Figure 3C). The linearity over the current densities measured suggests that the density of oxidized species drives OER under these conditions because of the change in the entropy of the oxidized species.^30,72−74^ These measurements are made in a regime where the density of oxidized species increases with the applied potential. At larger overpotentials, we expect the density of oxidized species to plateau, resulting in a deviation from a population-based model. We note that a recent study focused on IrO_*x*_ demonstrated that oxidized species drive water oxidation by reducing the activation energy of a chemical rate-determining step, resulting in a linear slope between the charge density and the logarithm of the OER current.^16^ A similar analysis on the materials investigated herein does not yield a linear slope, suggesting a different mechanism, and different dependence of the OER kinetics on the density of oxidized species, than that proposed for IrO_*x*_ (Figure S22). While we cannot exclusively discard a mechanism with an electrochemical rate-determining step or coverage-dependent change in activation energy such as that proposed for IrO_*x*_,^16^ we find that the rate-law model and interpretations derived from it fit our data best. The observation of the second-order kinetics implies that two oxidized Ni species (Ni^4+^–O) can chemically combine to form molecular oxygen. We note that in our previous work, we have reported a distinct fourth-order behavior from analogous analyses on electrodeposited Ni(*Fe*)OOH films,^30^ which we discuss further below. In order to rationalize the trends in the intrinsic TOF based on the spectroelectrochemical data, we propose a mechanism based on oxo coupling (Figure 4A). Following the Ni^2+^/Ni^3+^ redox transition, the oxyhydroxide formed consists of one *OH bound to the Ni^3+^ site. Upon increasing the potential (>1.5 V_RHE_), these *OH groups can further deprotonate to form *O species, as probed by an increase in differential absorption (Step 1). From the rate-law analysis, it can be concluded that two oxo species chemically combine to form molecular oxygen (Step 2), where the rate of reaction is dependent on the density of oxidized species. Finally, OH^–^ ions from the solution can adsorb on a Ni site to restore the active state of the catalyst (Step 3). Based on this mechanism, Step 1 can be assigned as the potential limiting step because it involves the electron transfer and is therefore promoted at higher potentials. On the other hand, Step 2 is the chemical rate-determining step that does not involve electron transfer. Using this mechanism, we can rationalize the volcano trend shown in Figure 3B. On the strong-binding side of the volcano, Mn-doping results in the Ni site binding *OH and *O stronger than is optimal, limiting the overall reaction at the step involving the combination of two oxo species and their removal (step 2). On the weak-binding side of the volcano, Zn-doping results in the Ni site binding *OH and *O weaker than is optimal, resulting in the potential-induced formation of *O species impeding the overall kinetics (step 1). In fact, for a given density of oxidized species, although the undoped oxide has a higher TOF and can produce a larger current density compared to the Mn-doped oxide (Figure 3C), at potentials lower than 1.7 V_RHE_, the density of oxidized species in the undoped oxide is negligible, resulting in lower water oxidation rates.

The potential limiting step proposed is consistent with previous theoretical models. Studies on a wide range of oxide materials have largely suggested that the second proton coupled electron-transfer step is the potential determining step (*OH + OH^–^ → *O + H_2_O + e^–^), with values of (Δ*G*_O2_ – Δ*G*_OH2_) ranging from ∼1.6 to 1.8 eV for doped NiO_*x*_, depending on the nature of the dopant and the active site.^25,31,32^ The theoretical coverage of oxidized species as a function of potential for a given (Δ*G*_O2_ – Δ*G*_OH2_) can be obtained using a model based on Langmuir electroadsorption, as shown in Figure 4B (details in the Methodology section). The earlier onset for the formation of oxidized species in the Mn-doped sample can be represented by Δ*G*_O2_ – Δ*G*_OH2_ = 1.56 eV, and the data for the Zn-doped sample can be represented by Δ*G*_O2_ – Δ*G*_OH2_ = 1.75 eV, in close agreement with that predicted using theoretical calculations on ideal doped NiOOH surfaces.^25,31,32^ Therefore, this analysis not only confirms that the potential determining step is the formation of the oxo species but also provides an experimental route to measure these adsorption free energies.

Considering these oxides bind *O weakly (Δ*G*_O2_ – Δ*G*_OH2_ > 1.58 eV), as validated by multiple theoretical studies^31^ in addition to the experimental results herein, the O–O bond formation between neighboring oxo groups to release molecular oxygen is a viable mechanism. Van Voorhis et al. have computed the barrier required for O–O bond formation on two adjacent sites in a Co_4_O_4_ cluster, and it was found to be ∼0.1 eV.^38^ Considering that the Co–Co bond distance in Co_4_O_4_ (∼2.8 Å) is comparable to the Ni–Ni bond distance in NiOOH,^25^ we do not expect geometric constraints to severely inhibit oxo coupling.

Because the focus in the literature has been largely placed on determining the potential determining step, partly due to the ease of computing the energetics, the mechanism for O–O bond formation has largely been elusive. Although binuclear mechanisms have been proposed in the literature for oxyhydroxide-based materials,^34−38^ experimental evidence, including its influence on the OER activity has been lacking so far. Here, the O–O bond formation is suggested to occur via a chemical combination of two neighboring oxo species, where the oxygen binding controls the formation of oxo species as well as their recombination and release. This sheds light on an important aspect of the mechanism, providing crucial insights into the factors controlling the intrinsic TOF. Based on this proposed mechanism, we hypothesize that the rate of water oxidation could not only be influenced by the binding energy of oxygenated intermediates but also by the atomic arrangement of active centers that can facilitate oxo coupling via geometrical effects.^75,76^ Interestingly, we find that on electrodeposited Ni(*Fe*)OOH films in our previous work, a fourth-order dependence on the density of oxidized species was observed,^30^ which can possibly be either due to geometric oxo coupling effects or due to different binding energetics on these surfaces, owing to the more amorphous structure of electrodeposited films compared to the as-synthesized crystalline nanoparticles investigated herein. Although our Raman results point to the formation of oxyhydroxide phases, the crystalline oxide core of the nanoparticles might impact the local geometric and electronic structure of the active site. Further work needs to be undertaken to directly link the active site environment to the differences in the reaction order observed. Using this new insight into the reaction mechanism, it would be possible to rationally design water oxidation catalysts that can exhibit an intrinsic TOF that outperforms the current state-of-the-art catalysts.

## Conclusions

In summary, in this work, we have directly probed the density of oxidized species as a function of potential in the OER regime and determined their role in catalyzing the water oxidation reaction on metal-doped NiO. Using optical *operando* spectroscopy, coupled with time-resolved stepped potential spectroelectrochemistry, we deduce that doping NiO can influence the density of oxidized species formed at a given potential, with the greatest density generated in Mn-doped, followed by Co-, Fe-, undoped, and Zn-doped NiO_*x*_ because of the alteration in the energetics of the oxygen binding energy on the Ni site. This allows us to quantitatively define the intrinsic TOF, which increases from Mn- to Co- to the Fe-doped sample and then decreases for the undoped and Zn-doped samples. Based on the rate-law analysis, we find that the OER current follows a second-order behavior with respect to the oxidized Ni sites, suggesting a mechanism involving oxo–oxo coupling on two neighboring sites. Consequently, we propose that for Ni centers that bind oxygen too strongly (Mn- and Co-doped), the rate-determining step of O–O coupling and O_2_ removal impedes the overall reaction rate, whereas for the Ni centers that bind oxygen too weakly (undoped and Zn-doped), the potential determining step of *O formation from *OH controls the rate of reaction. By correlating experimentally determined binding energetics from the redox peak centers to the intrinsic TOF, we elucidate the role of binding energetics in controlling the rate of reaction. This study thus experimentally demonstrates the Sabatier principle of achieving optimal binding energetics for the highest kinetics. Based on the volcano relation observed in our study, we propose that novel catalysts with binding energetics slightly weaker than Fe_0.1_Ni_0.9_O, but stronger than undoped NiO, will have a higher TOF than those reported for Fe_0.1_Ni_0.9_O herein. This study also highlights the role of chemical rate-determining steps and cooperative effects between oxidized states in governing the reaction rates of electrochemical reactions and thus advances our understanding of the role of oxidized species in facilitating O–O bond formation during water oxidation.

## Methods

### Synthesis

Two equimolar solutions (0.5 M) of Ni(NO_3_)_2_ and M^*n*+^Cl_*n*_ were separately prepared. Ethylene glycol (EG) was added to the Ni solution to obtain a 1:0.95 metal fuel ratio. Finally, the Ni and EG solution was mixed with the dopant one in a 9:1 ratio to keep the final metal concentration at 0.5 M. The new solution was placed in a porcelain crucible and allowed to stir for 1 h before transferring it into a preheated muffle furnace at 250 °C during 30 min for the combustion.

### Powder X-ray Diffraction

PXRD patterns of NiO and M-NiO powder samples were recorded on a D8 Advance Series 2Theta/Theta powder diffraction system using CuKα1-radiation in the transmission geometry. The system is equipped with a VÅNTEC-1 single-photon counting PSD, a Germanium monochromator, a ninety position auto changer sample stage, fixed divergence slits, and a radial soller. The angular 2θ diffraction range was between 5° and 70°. The data was collected with an angular step of 0.02° at 12 s per step and sample rotation.

### Scanning Electron Microscopy

The ESEM instrument is from FEI company, model Quanta 600 in low vacuum mode (vacuum pressure 0.68 Torr). The energy-dispersive X-ray (EDX) instrument is from Oxford Instruments. The conditions for ESEM are 20 kV accelerating voltage and a working distance close to 10 mm.

### Transmission Electron Microscopy

TEM images were collected using a JEOL 1011 transmission electron microscope operating at 80 kV. Samples were dispersed in ethanol, and a drop of resultant suspensions was poured on carbon-coated copper grids.

### Electrode Preparation

The as-prepared catalysts (5 mg) were dispersed in 1 mL of a solution of 987 μL of EtOH/H_2_O (3/1) and 12.7 μL of FAA Fumatech anionomer (ca. 10% w/w respect to the catalyst). Four coatings of 125 μL each (for a total of 500 μL) of such a dispersion were sprayed with an airbrush onto an FTO glass slide, whose surface was entirely covered by a Kapton tape, except an exposed area of 10 × 10 mm^2^. The resultant loading is 2.5 mg/cm^2^. To ensure a fast deposition and homogeneous film, the FTO was placed on a hot plate at 75 °C during the entire process. Before deposition, FTO slides have been washed by sonication in HCl concentrated, ethanol, and acetone (10 min, respectively).

### Electrochemistry

Electrochemical measurements were performed using an Autolab potentiostat (PGSTAT 101) in a three-electrode configuration with a Pt mesh as the counter electrode and an Ag/AgCl (saturated KCl) as the reference electrode. The standard potential of the reference electrode was calibrated against the reversible hydrogen electrode constructed using a Pt wire immersed in the electrolyte saturated with hydrogen gas. Impedance spectroscopy was used to determine the uncompensated series resistance (∼30 to ∼50 Ω), and measurements were made at OER-relevant potentials from 0.1 MHz to 1 Hz. All measurements were performed in the purified Fe-free electrode in accordance with the procedure described by Trotochaud et al.^8^

### Spectroelectrochemistry

Optical absorption as a function of potential was determined by fitting a spectroelectrochemical cell in a Cary 60 UV–vis spectrometer (Agilent Technologies). Measurements were made under potentiostatic conditions, and spectra were collected after the current stabilized. Stepped potential spectroelectrochemical measurements rely on a potential jump (pump) and an optical probe.^30^ Upon applying a potential, change in optical absorption was monitored using light from a 100 W tungsten lamp (Bentham IL1), with an Oriel cornerstone 130 monochromator. The transmitted light was filtered by several band pass and long pass filters (Comar Optics) and detected using a silicon photodiode (Hamamatsu S3071). The photons were converted to a voltage signal, which was passed through an amplifier (Costronics) and recorded using an oscilloscope (Tektronics TDS 2021c) and with a DAQ card (National Instruments, NI USB06211). The time resolution is ms–s. A Palmsens3 potentiostat was used. All optical and electrochemical data were acquired using a home-built LabView software.

### Raman Spectroscopy

*Operando* vibrational (Raman) spectroscopy measurements were performed with a WITec Apyron confocal microscope using a 532 nm laser with power in the range 10–30 mW. A grating of 2400 g/mm and BLZ = 500 nm were used with an optical objective Zeiss LD EC Epiplan-Neofluar Dic 50×/0.55. In situ and *operando* analyses were carried out using a commercial three-electrode Raman cell (redox.me) with a Pt wire and Ag/AgCl (3 M KCl) as counter and reference electrodes, respectively.

### Modeling Coverage of Oxidized Species

Langmuir electroadsorption is based on the following assumptions: (1) all sites have the same adsorption energetics, (2) one adsorbate is present per site, and (3) there is no lateral interaction between adsorbates.^77,78^ The hydroxide-mediated deprotonation, which occurs at OER-relevant potentials can be simply written as *OH + OH^–^ → *O + H_2_O + e^–^. The Gibbs free energy for this reaction is Δ*G =* ΔG_*O_*+* Δ*G*_H2O_ – *U* – ΔG_*OH_ – Δ*G*_OH–_, where the activity of H_2_O and OH^–^ is assumed to be 1. The coverage of oxidized sites, θ_O_, with respect to hydroxylated sites, θ_OH_, is given by θ_O_= K*θ_OH_, where *K* is the equilibrium constant, which depends on Δ*G* via *K =* exp(−Δ*G*/*RT*). Furthermore, the sum of sites is conserved, that is, each Ni center can either be occupied by *OH or *O: θ_OH_*+* θ_O_*=* 1. This results in θ_O_*= K*/(1 *+ K*). Therefore, for a given value of Δ*G*_O_*–* Δ*G*_OH_, the coverage can be deduced as a function of potential.

## Supporting Information Available

Additional sample characterization (XRD, SEM, Raman spectroscopy, electrochemistry), operando Raman and UV–vis spectroscopy data, including stepped potential spectroelectrochemistry, and Tafel plots (PDF)
