# Supplementary material for: Spectroelectrochemical Analysis of the Water Oxidation Mechanism on Doped Nickel Oxides
Source: J Am Chem Soc. 2022 Apr 20;144(17):7622–33. doi: 10.1021/jacs.1c08152 (PMC9073940; doi:10.1021/jacs.1c08152)
Supplement: Supplementary file 1 — ja1c08152_si_001.pdf [file ja1c08152_si_001.pdf]

Supplementary Information

Spectroelectrochemical analysis of the water oxidation mechanism on doped nickel oxides

Reshma R Rao* [1, 6], Sacha Corby[1, 6], Alberto Bucci[2], Miguel García-Tecedor[3], Camilo A. Mesa[3], Jan Rossmeisl[4], Sixto Giménez[3], Julio Lloret-Fillol[2], Ifan E.L. Stephens[5], James R. Durrant* [1]

[1]Department of Chemistry, Centre for Processable Electronics, Imperial College London, London W12 0BZ, United Kingdom
[2]Institute of Chemical Research of Catalonia (ICIQ), The Barcelona Institute of Science and Technology, Avinguda Països Catalans 16, 43007 Tarragona, Spain
[3]Insitute of Advanced Materials (INAM), University Jaume I, 12071 Castello de la Plana, Spain
[4]Department of Chemistry, University of Copenhagen, Universitetsparken 5, Copenhagen DK-2100, Denmark
[5]Department of Materials, Royal School of Mines, Imperial College London, South Kensington Campus, London SW7 2AZ, U.K.

[6] These authors contributed equally

Email: reshma.rao@imperial.ac.uk, j.durrant@imperial.ac.uk

X-Ray Diffraction

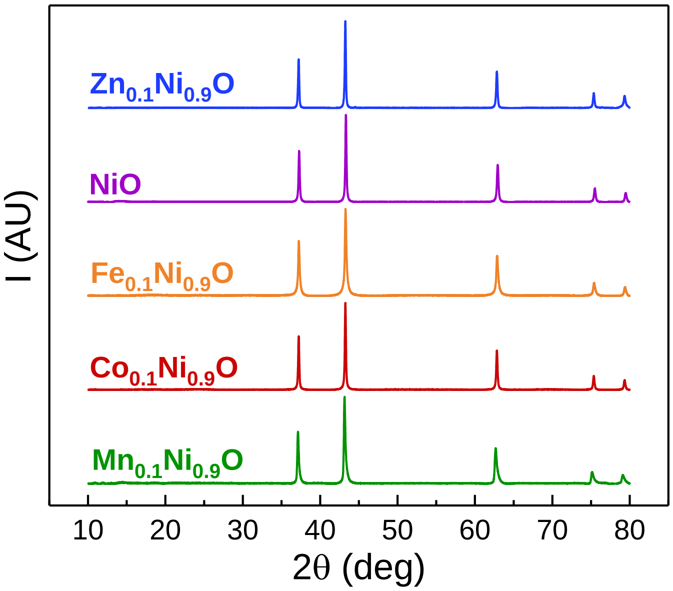

Figure S1: PXRD spectra of NiO (purple) and $M_{0.1}Ni_{0.9}O$ (green = Mn, red = Co, orange = Fe, blue = Zn, purple = undoped).

Scanning Electron Microscopy

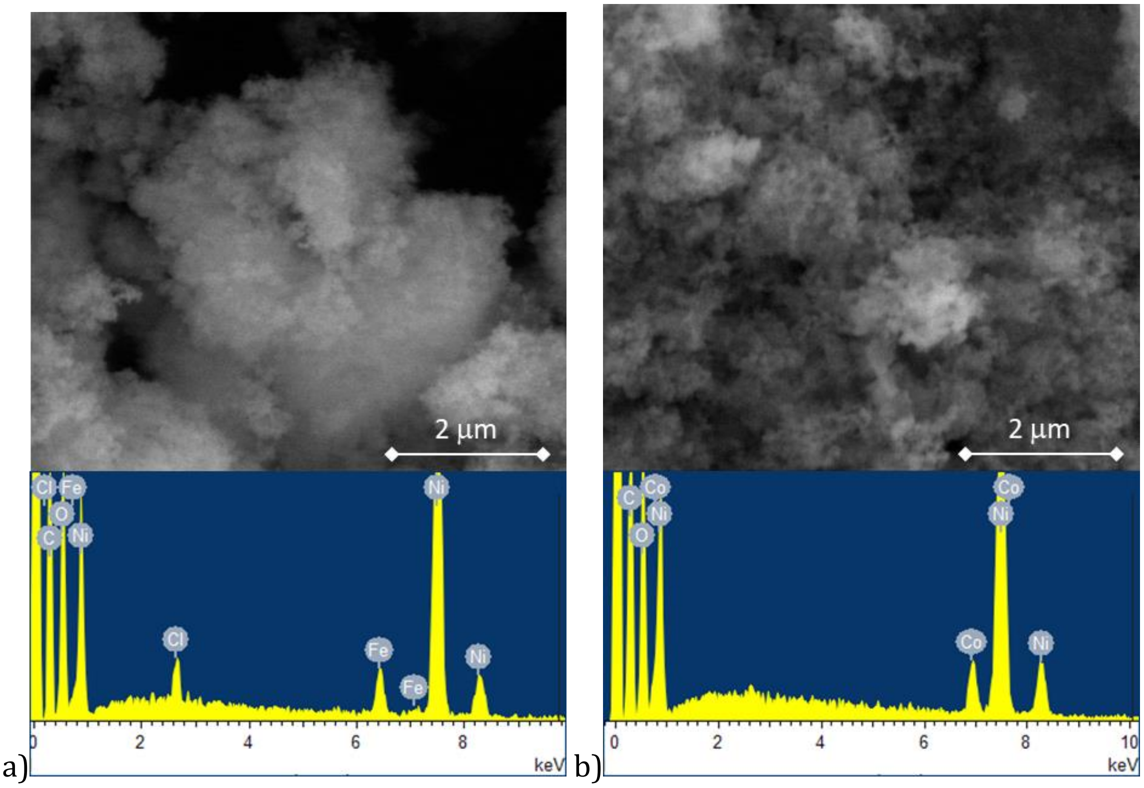

a) b)

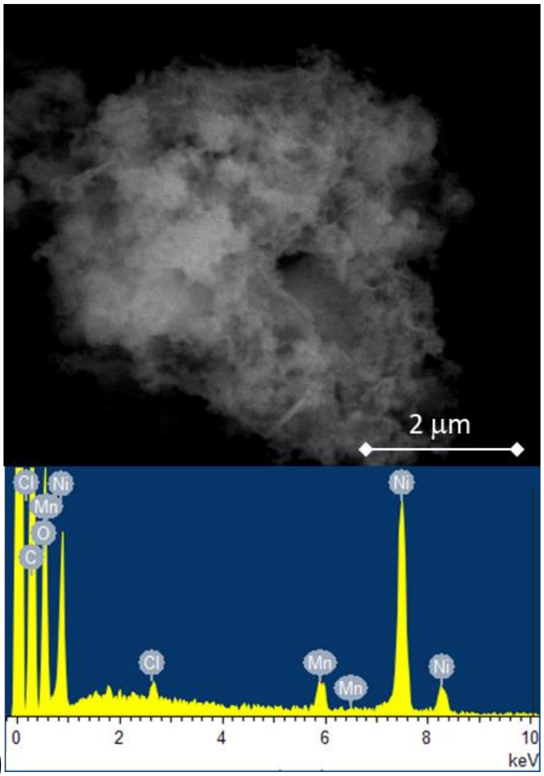

c)
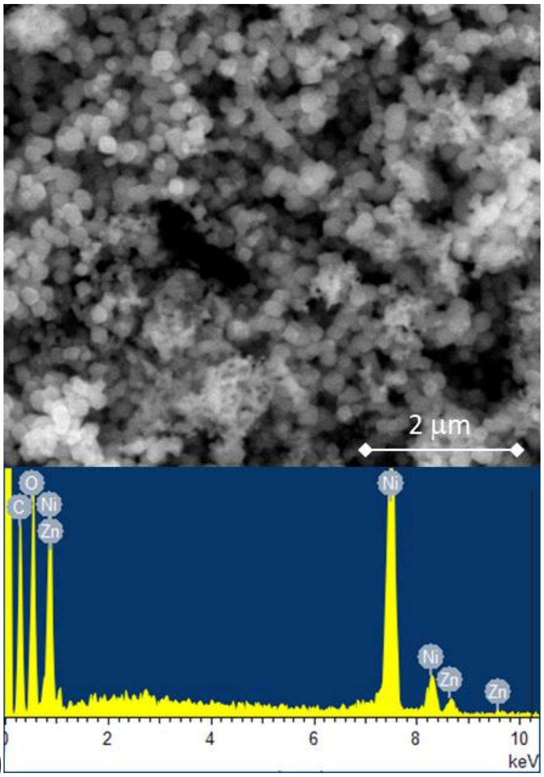

d)

Figure S2: ESEM images (top) with corresponding EDX spectrum (bottom) for (a) $Fe_{0.1}Ni_{0.9}O$, (b) $Co_{0.1}Ni_{0.9}O$, (c) $Mn_{0.1}Ni_{0.9}O$, and (d) $Zn_{0.1}Ni_{0.9}O$.

Transmission Electron Microscopy

a)
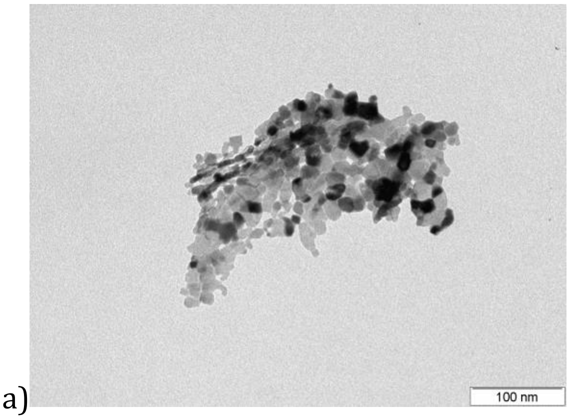

b)
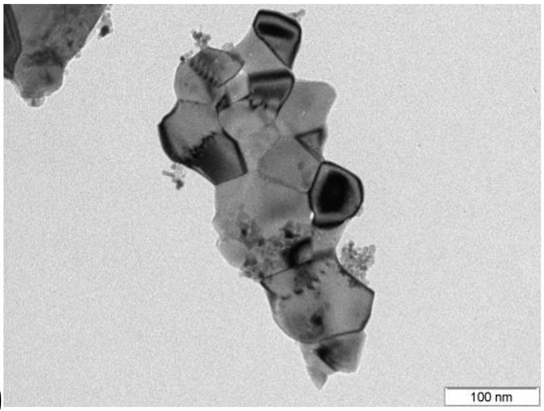

c) 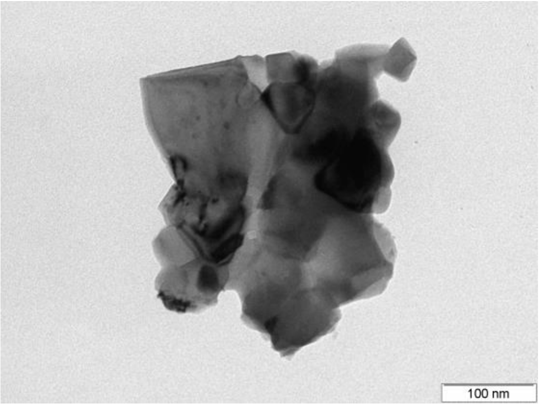 d) 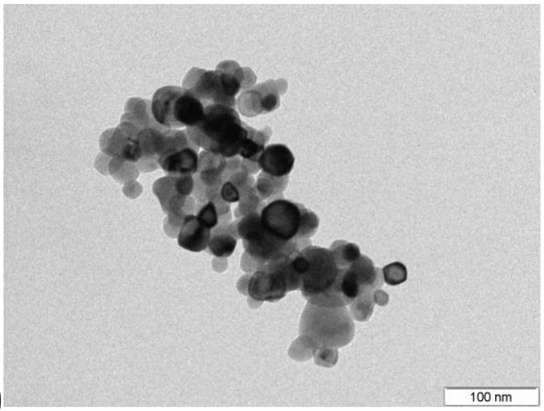

Figure S3: TEM images of (a) $Fe_{0.1}Ni_{0.9}O$, (b) $Co_{0.1}Ni_{0.9}O$, (c) $Mn_{0.1}Ni_{0.9}O$, and (d) $Zn_{0.1}Ni_{0.9}O$.

Table S1: Particle sizes obtained from TEM for the samples investigated in this study

Sample	TEM mean particle size
NiO	70
$Fe_{0.1}Ni_{0.9}O$	21
$Co_{0.1}Ni_{0.9}O$	31
$Mn_{0.1}Ni_{0.9}O$	14
$Zn_{0.1}Ni_{0.9}O$	47

Raman Spectroscopy

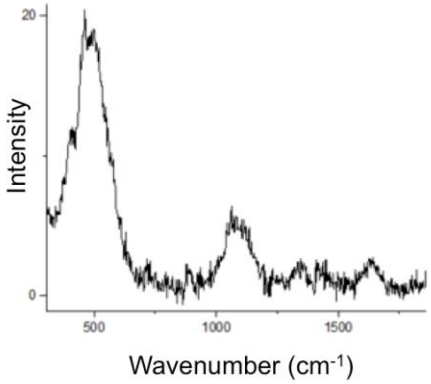

Figure S4: Raman spectra of an undoped NiO at open circuit. The peaks present at ~1100 cm^{-1} and ~1650 cm^{-1} confirm the presence of the rocksalt phase

.

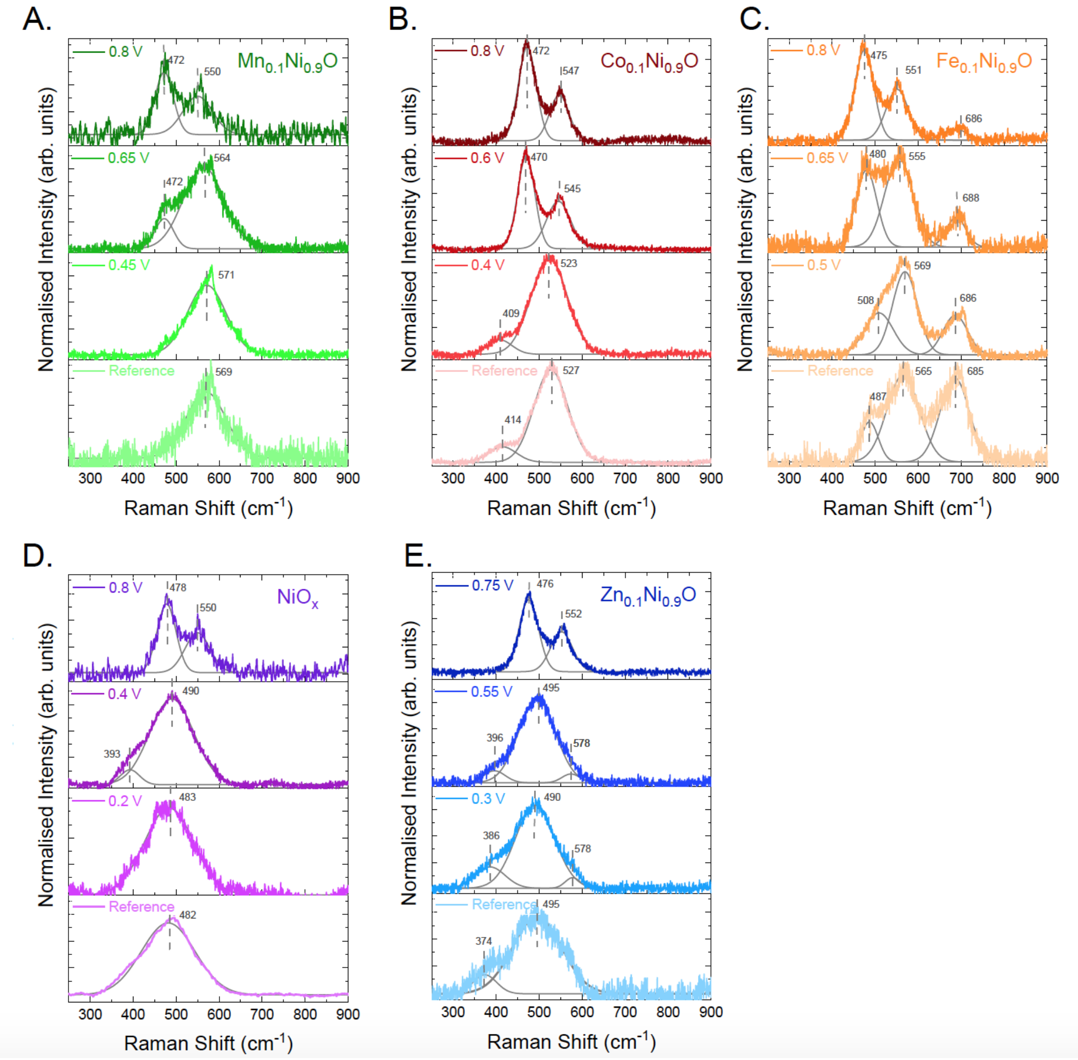

Figure S5: Raman spectra as a function of potential for (A) Mn-doped (B) Co-doped (C) Fe-doped (D) undoped and (E) Zn-doped NiO. Increasing potential leads to the formation

of the oxyhydroxide phase. The measurements were done in 0.1 M KOH. Reference refers to open circuit potential conditions before any potential is applied. The potentials are referenced relative to an Ag/AgCl reference electrode.

Ni^{2+}/Ni^{3+} redox peak position

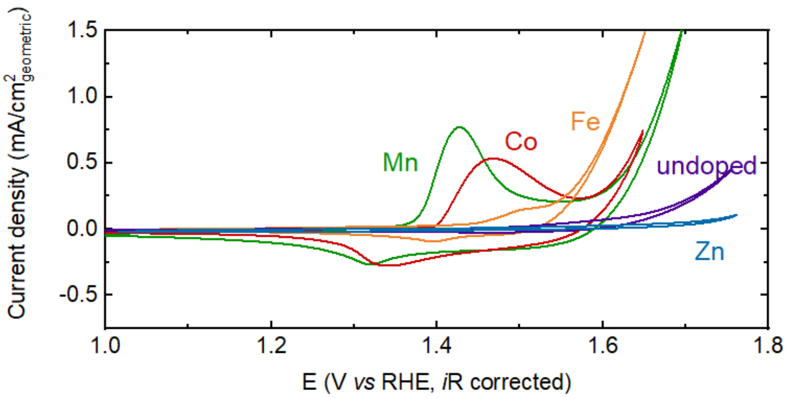

Figure S6: Cyclic voltammograms of the Mn-, Co- and Fe- Zn- and undoped NiO. All measurements were made in Fe-free 0.1 M KOH on samples deposited on FTO at 10 mV/s.

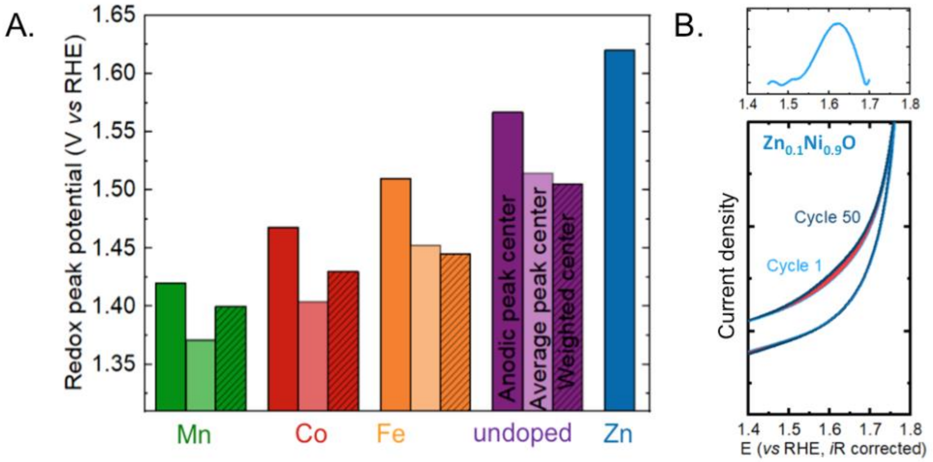

Figure S7: (A) Summary for all redox peak potentials. The anodic peak center is shown in dark shaded color, the average peak center is shown in light shaded color and the weighted average of the anodic and cathodic redox peak center is shown in hatched bars. (B) Cyclic voltammetry for the 1st and 50th scan of the Zn-doped NiO shows the growth of the redox feature at ~1.6 V_{RHE}. The top panel shows the difference in current density between these 2 cycles.

The weighted redox peak of the oxidation and reduction peak[1] has been defined as: $V_{weighted} = \frac{\int V\, dQ}{\int dQ}$, after correcting for the double-layer capacitance. Here Q is the charge transferred at voltage V in the redox process. The weighted centre is defined as the average value of the oxidation and reduction weighted centres.

***Operando* spectroscopy**

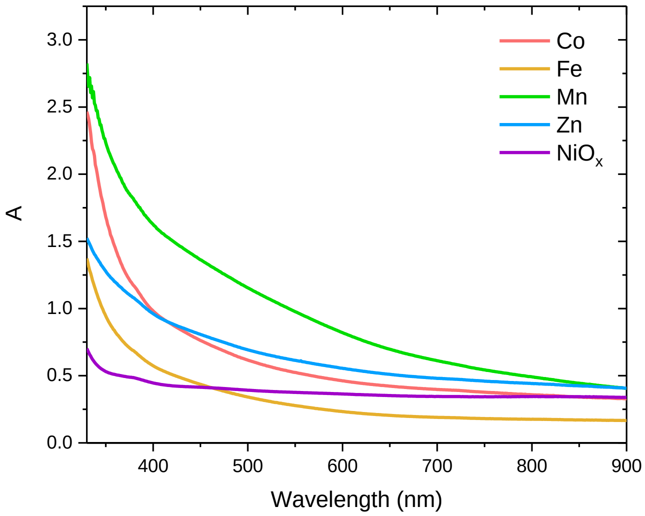

Figure S8: UV-Vis absorbance spectra for all catalysts before activation, measured under open circuit potential in 0.1 M KOH.

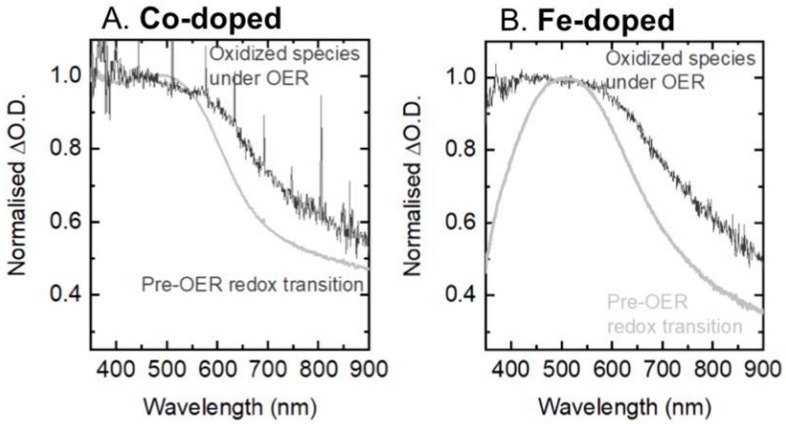

Figure S9: Normalized differential absorption for the species corresponding to the pre-OER redox transition (grey) and oxidized species present at OER potentials (black) for the (A) Co-doped NiO and (B) Fe-doped NiO.

The optical spectroscopy data shows two distinct features:

- A sharp feature centred at ~500 nm corresponding to the redox transition prior to OER.
- A broader feature with a peak from ~350 – 600 nm corresponding to the species that accumulate during OER.

The low signal-noise ratio for the Zn-doped and undoped sample made the distinction of these two different features difficult.

The operando spectroelectrochemistry data as a function of potential is generally presented as difference spectra (ΔO.D), which are obtained by subtracting the reference spectrum from the spectra collected at the potential of interest. The reference spectrum

has been selected as the OCP value measured a few seconds after activation as detailed in our previous work[2].

We note that the broad absorption bands are consistent with literature reports on similar materials[2,3,4,5]. We detect negligible counts on the detector during diffuse reflectance measurements relative to a 100% reflecting sample suggesting that negligible light is reflected from the sample. No changes in signal are observed as a function of potential on a representative Mn-NiO sample from 1.50 V_{RHE} to 1.60 V_{RHE}, demonstrating no potential-induced changes in scattering. Therefore, changes observed in the spectra can be attributed to changes in optical absorption due to formation of oxidized Ni states.

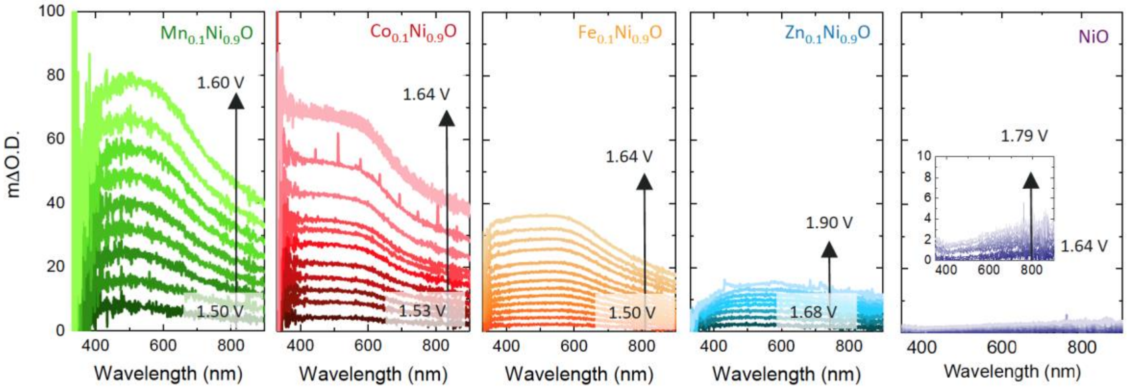

Figure S10: Differential UV-vis spectra as a function of potential (noted in the figure) for the Mn-doped (green), Co-doped (red), Fe-doped (yellow), Zn-doped (blue) and undoped (purple) NiO.

Open circuit decay measurements

In order to determine the density of oxidized species, stepped potential spectroelectrochemistry measurements were performed. The charge extracted upon applying a less positive potential was correlated to the optical signal. This analysis requires assuming that upon applying a less oxidizing potential, the electrode discharges via passage of electrons through the external circuit as opposed to discharge via water oxidation. This assumption can be verified by comparing the time constant for charge extraction via the external circuit to the time constant for water oxidation. As seen in Figure 2c, the time scale for charge extraction and optical absorption decay is ~1 second. In contrast, the time scale of water oxidation can be obtained by open circuit decay measurements after exposing the electrode to water oxidation potentials. During open circuit decay measurements charge cannot be extracted through the external circuit and the electrode must discharge via the oxidation of water as has been reported previously[2, 6,]. This time scale of water oxidation obtained using open circuit decay measurements (Figure S11, S12) is 5-10 times slower than that of charge extraction through the film (Figure 2c). Its longer time constant thus shows that upon application of a reducing potential as has been done during the stepped potential measurements in Figure 2c, the oxidized species are more likely to be reduced by charge extraction through the external circuit as opposed to reducing by oxidizing of water.

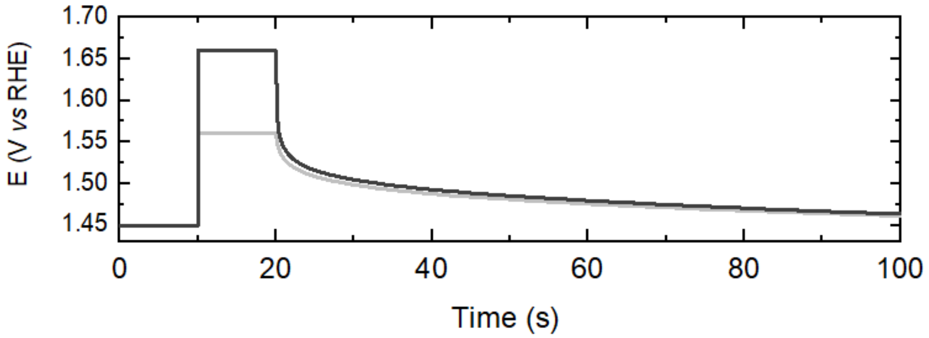

Figure S11: (A) Measurement showing the potential decay as a function of time for $Fe_{0.1}Ni_{0.9}O$ For the first 10 seconds, a potential of 1.45 V_{RHE} was applied. Between 10 and 20 seconds, a potential of 1.56 V_{RHE} (grey trace) and 1.66 V_{RHE} (black trace) was applied. After 20 seconds, the measurement configuration was switched to open circuit (zero current density) and the decay in optical signal was recorded.

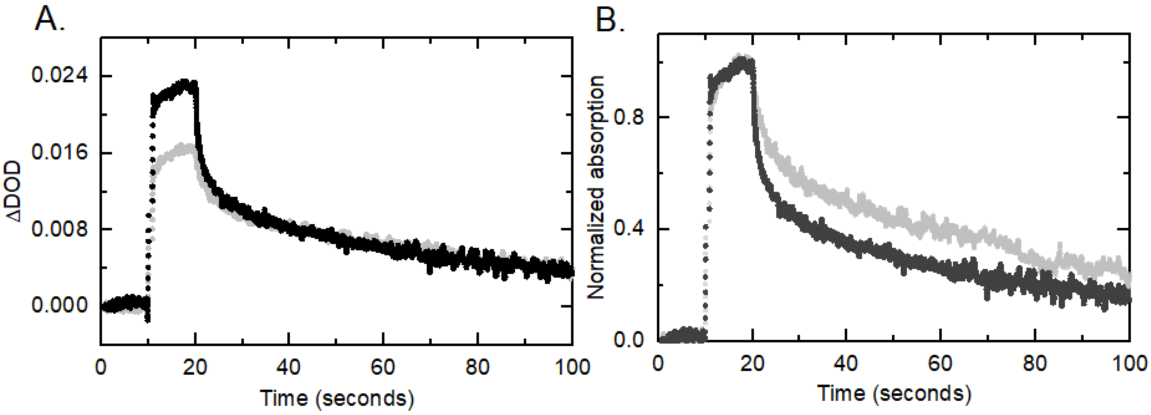

Figure S12: (A) Measurement showing the optical signal decay as a function of time for $Fe_{0.1}Ni_{0.9}O$ at a wavelength of 500 nm (B) Normalized optical signal decay as a function of time for $Fe_{0.1}Ni_{0.9}O$. For the first 10 seconds, a potential of 1.45 V_{RHE} was applied. Between 10 and 20 seconds, a potential of 1.56 V_{RHE} (grey trace) and 1.66 V_{RHE} (black trace) was applied. After 20 seconds, the measurement configuration was switched to open circuit (zero current density) and the decay in optical signal was recorded. The time constant for the fast decay provides insight into the lifetime of the active species.

Calculation of extinction coefficient

The extinction coefficient was calculated using the stepped potential spectroelectrochemistry method. A voltage pulse was applied, and the corresponding change in optical absorption and current density were measured simultaneously. The measured optical data is proportional to the density of oxidized species in the sample. Upon switching the potential back to the lower value, a reductive spike in the current is observed, which corresponds to the reduction in the oxidized species. The current-time response during the reductive spike can be integrated to deduce the charge corresponding to reduction in the oxidized states. We note that the response time from the optical

measurements is in good agreement with the response time of the charge accumulation in the oxide, as shown in Figure S13. Using the Lambert-Beer Law, the extinction coefficient can be extracted by plotting the optical signals as a function of the charge. The slope of the graph yields the extinction coefficient.

Lambert-Beer Law:

$$A = \varepsilon * c$$

where:
A = absorbance at a given wavelength,
ε = extinction coefficient
c = concentration of electrons per cm^2

These measurements were made at a wavelength of 500 nm, where the optical intensity is maximum.

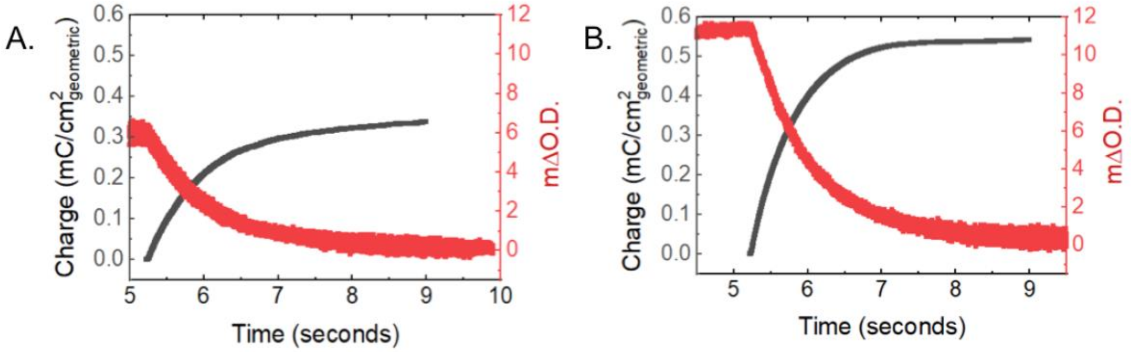

Figure S13: Change in optical absorption (red) and absolute value for change in accumulated charge (grey) as a function of time during a potential step from (A) 1.525 V_{RHE} to 1.515 V_{RHE} and (B) 1.54 V_{RHE} to 1.515 V_{RHE} for a Mn-doped NiO sample.

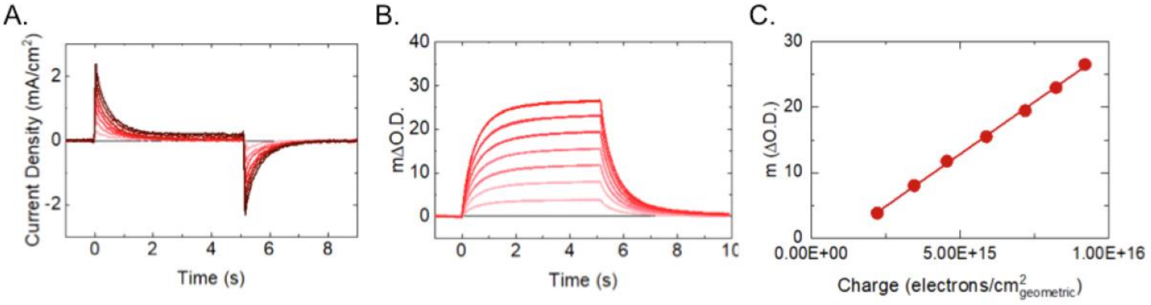

Figure S14: Calculation for Co-NiO deposited on FTO. (A) electrochemical data and (B) optical data obtained from stepped voltage absorption spectroscopy for increasingly larger potential steps in the OER region. (C) Extinction coefficient obtained from gradient of optical signal to charge. Measurements were made in 0.1 M KOH.

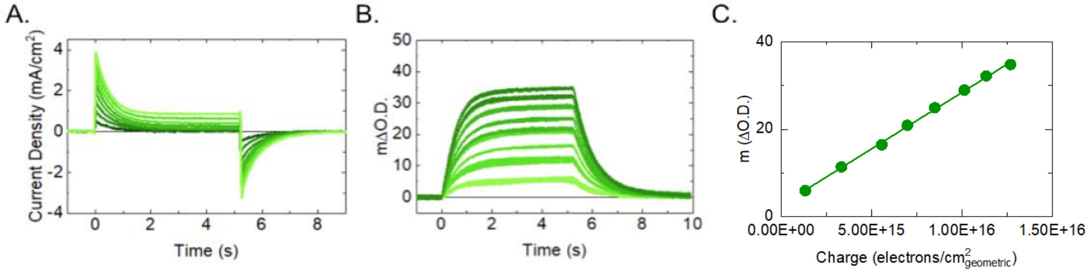

Figure S15: Calculation for Mn-NiO deposited on FTO. (A) electrochemical data and (B) optical data obtained from stepped voltage absorption spectroscopy for increasingly larger potential steps in the OER region. (C) Extinction coefficient obtained from gradient of optical signal to charge. Measurements were made in 0.1 M KOH

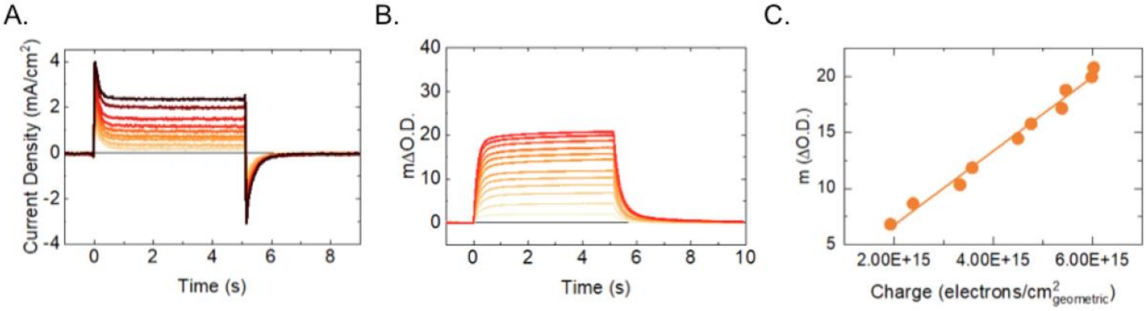

Figure S16: Calculation for Fe-NiO deposited on FTO. (A) electrochemical data and (B) optical data obtained from stepped voltage absorption spectroscopy for increasingly larger potential steps in the OER region. (C) Extinction coefficient obtained from gradient of optical signal to charge. Measurements were made in 0.1 M KOH

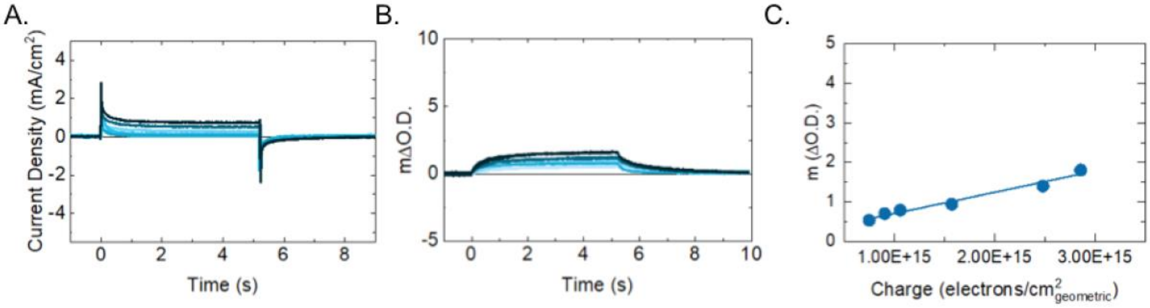

Figure S17: Calculation for Zn-NiO deposited on FTO. (A) electrochemical data and (B) optical data obtained from stepped voltage absorption spectroscopy for increasingly larger potential steps in the OER region. (C) Extinction coefficient obtained from gradient of optical signal to charge. Measurements were made in 0.1 M KOH

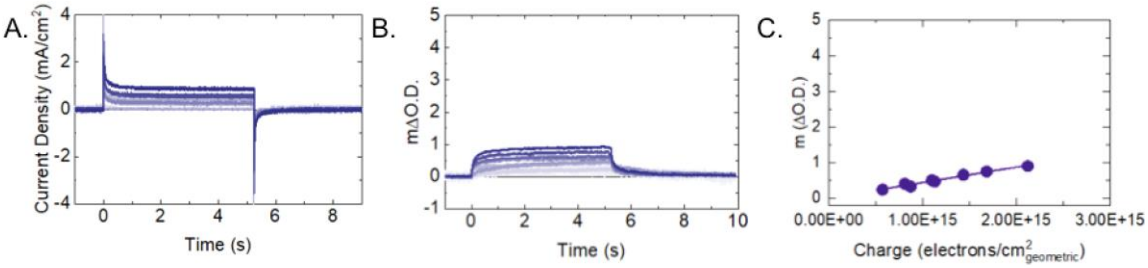

Figure S18: Calculation for NiO deposited on FTO. (A) electrochemical data and (B) optical data obtained from stepped voltage absorption spectroscopy for increasingly larger potential steps in the OER region. (C) Extinction coefficient obtained from gradient of optical signal to charge. Measurements were made in 0.1 M KOH

Approximation of the fraction of oxidized states

The loading of oxide on the FTO substrates is ~2.5 mg/cm^2.

2.5 mg of NiO corresponds to ~3*10^{-5} moles of NiO. This is equivalent to ~2*10^{19} Ni sites per cm^2 or 200000 Ni sites per nm^2.

Therefore, the fraction of oxidized states generated correspond to:

$$\frac{\text{Density of oxidized states per nm}^2}{200000\ \text{Ni sites per nm}^2}$$

For 100 oxidized sites per nm^2 (corresponding to the species density at ~1.52 V_{RHE}, ~1.58 V_{RHE}, ~1.62 V_{RHE}, ~1.73 V_{RHE} and ~1.80 V_{RHE} for the Mn-, Co-, Fe-, Zn- and undoped samples respectively), the fraction of oxidized metal sites would be on the order of ~0.1% of the total.

Tafel Plots

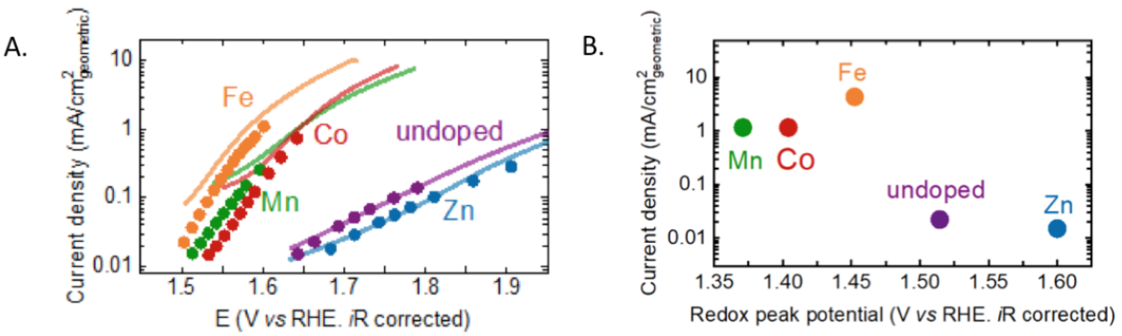

Figure S19: (A) OER activity defined as current density normalized to the geometric surface area. Lines show data from the capacitance-corrected cyclic voltammograms and points in the corresponding color show data from potentiostatic measurements. (B) Normalized current density measured at 1.65 V_{RHE} as a function of the redox peak potential of the pre-OER redox peak

pH Dependence

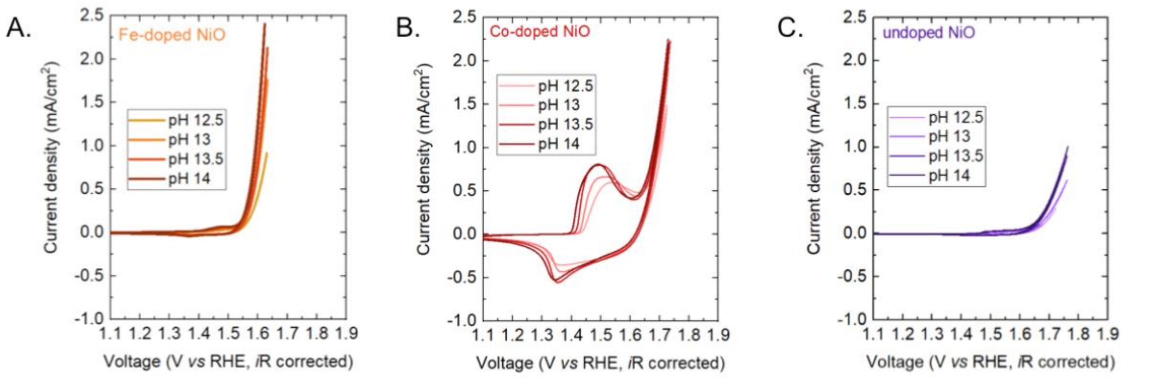

Figure S20: pH-dependent measurements of OER activity from pH 12.5 to pH 14 for (A) Fe-doped (B) Co-doped and (C) undoped NiO

The reaction order with respect to the concentration of OH^- ions can be determined by obtaining the slope of the logarithm of the current at a constant potential on the reversible hydrogen electrode scale as a function of the logarithm of the concentration of OH^- ions, as shown in Figure S21. Reaction orders of less than 0.3 indicate a very weak pH dependence.

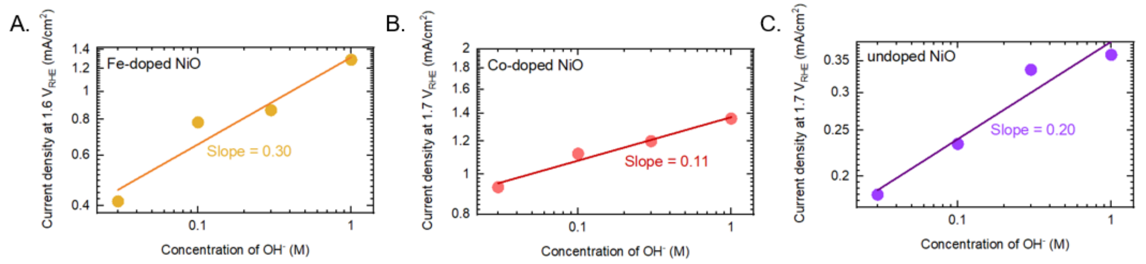

Figure S21: Reaction order of OH^- ions obtained from pH dependent measurements for (A) Fe-doped (B) Co-doped and (C) undoped NiO

Alternative model relating current density to density of oxidized species

We note that a recent report on IrO_x catalysts proposed a kinetic model relating the current density to the density of oxidized species[7]. In their proposed mechanism, the density of oxidized species reduces the activation energy of a chemical rate-determining step due to long-range effects:

$$i \propto \exp\left(\frac{-\zeta\theta + \kappa}{k_B T}\right)$$

where i is the current, ζ and κ are constants, θ is the coverage of oxidized species, k_B is Boltzmann's constant and T is the temperature. Consequently, according to this model, a linear slope is expected between the logarithm of the current density and the density of oxidized species. However, according to Figure S22, we do not find such a linear correlation.

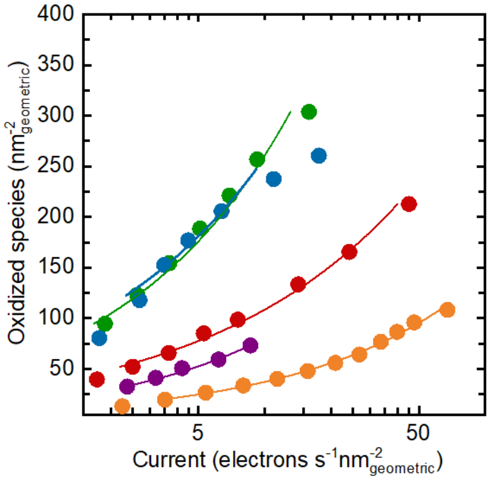

Figure S22: Density of oxidized species as a function of the current density for the Mn-(green), Co-(red), Fe-(orange), undoped (purple) and Zn-doped oxides. Note that the current has been plotted on a logarithmic scale.

References

(1) Kuznetsov, D. A.; Han, B.; Yu, Y.; Rao, R. R.; Hwang, J.; Román-Leshkov, Y.; Shao-Horn, Y. Tuning Redox Transitions via Inductive Effect in Metal Oxides and Complexes, and Implications in Oxygen Electrocatalysis. *Joule* **2018**, *2* (2), 225–244. https://doi.org/10.1016/j.joule.2017.11.014.

(2) Francàs, L.; Corby, S.; Selim, S.; Lee, D.; Mesa, C. A.; Godin, R.; Pastor, E.; Stephens, I. E. L.; Choi, K.-S.; Durrant, J. R. Spectroelectrochemical Study of Water Oxidation on Nickel and Iron Oxyhydroxide Electrocatalysts. *Nat. Commun.* **2019**, *10* (1), 5208. https://doi.org/10.1038/s41467-019-13061-0.

(3) Corby, S.; Tecedor, M.-G.; Tengeler, S.; Steinert, C.; Moss, B.; A. Mesa, C.; F. Heiba, H.; A. Wilson, A.; Kaiser, B.; Jaegermann, W.; Francàs, L.; Gimenez, S.; R. Durrant, J. Separating Bulk and Surface Processes in NiO x Electrocatalysts for Water Oxidation. *Sustain. Energy Fuels* **2020**, *4* (10), 5024–5030. https://doi.org/10.1039/D0SE00977F.

(4) Görlin, M.; Ferreira de Araújo, J.; Schmies, H.; Bernsmeier, D.; Dresp, S.; Gliech, M.; Jusys, Z.; Chernev, P.; Kraehnert, R.; Dau, H.; Strasser, P. Tracking catalyst redox states and reaction dynamics in Ni–Fe oxyhydroxide oxygen evolution reaction electrocatalysts: the role of catalyst support and electrolyte pH. *J. Am. Chem. Soc.* **2017**, *139* (5), 2070–2082. https://doi.org/10.1021/jacs.6b12250

(5) Goldsmith, Z. K.; Harshan, A. K.; Gerken, J. B.; Vörös, M.; Galli, G.; Stahl, S. S.; Hammes-Schiffer, S. Characterization of NiFe Oxyhydroxide Electrocatalysts by Integrated Electronic Structure Calculations and Spectroelectrochemistry. *Proc. Natl. Acad. Sci.* **2017**, *114* (12), 3050–3055. https://doi.org/10.1073/pnas.1702081114.
(6) Conway, B. E.; Bourgault, P. L. THE ELECTROCHEMICAL BEHAVIOR OF THE NICKEL – NICKEL OXIDE ELECTRODE: PART I. KINETICS OF SELF-DISCHARGE. *Can. J. Chem.* **1959**, *37* (1), 292–307. https://doi.org/10.1139/v59-038.
(7) Nong, H. N.; Falling, L. J.; Bergmann, A.; Klingenhof, M.; Tran, H. P.; Spöri, C.; Mom, R.; Timoshenko, J.; Zichittella, G.; Knop-Gericke, A.; Piccinin, S.; Pérez-Ramírez, J.; Cuenya, B. R.; Schlögl, R.; Strasser, P.; Teschner, D.; Jones, T. E. Key Role of Chemistry versus Bias in Electrocatalytic Oxygen Evolution. *Nature* **2020**, *587* (7834), 408–413. https://doi.org/10.1038/s41586-020-2908-2.
